# TCF7L2 Modulates Glucose Homeostasis by Regulating CREB- and FoxO1-Dependent Transcriptional Pathway in the Liver

**DOI:** 10.1371/journal.pgen.1002986

**Published:** 2012-09-27

**Authors:** Kyoung-Jin Oh, Jinyoung Park, Su Sung Kim, Hyunhee Oh, Cheol Soo Choi, Seung-Hoi Koo

**Affiliations:** 1 Division of Biochemistry and Molecular Biology, Department of Molecular Cell Biology and Samsung Biomedical Institute, Sungkyunkwan University School of Medicine, Suwon, Gyeonggi-do, Korea; 2 Korea Mouse Metabolic Phenotyping Center, Lee Gil Ya Cancer and Diabetes Institute, Gil Medical Center, Gachon University, Yeonsu-ku, Incheon, Korea; Dana-Farber Cancer Institute, United States of America

## Abstract

Peripheral insulin resistance contributes to the development of type 2 diabetes. TCF7L2 has been tightly associated with this disease, although the exact mechanism was largely elusive. Here we propose a novel role of TCF7L2 in hepatic glucose metabolism in mammals. Expression of medium and short isoforms of TCF7L2 was greatly diminished in livers of diet-induced and genetic mouse models of insulin resistance, prompting us to delineate the functional role of these isoforms in hepatic glucose metabolism. Knockdown of hepatic TCF7L2 promoted increased blood glucose levels and glucose intolerance with increased gluconeogenic gene expression in wild-type mice, in accordance with the PCR array data showing that only the gluconeogenic pathway is specifically up-regulated upon depletion of hepatic TCF7L2. Conversely, overexpression of a nuclear isoform of TCF7L2 in high-fat diet-fed mice ameliorated hyperglycemia with improved glucose tolerance, suggesting a role of this factor in hepatic glucose metabolism. Indeed, we observed a binding of TCF7L2 to promoters of gluconeogenic genes; and expression of TCF7L2 inhibited adjacent promoter occupancies of CREB, CRTC2, and FoxO1, critical transcriptional modules in hepatic gluconeogenesis, to disrupt target gene transcription. Finally, haploinsufficiency of TCF7L2 in mice displayed higher glucose levels and impaired glucose tolerance, which were rescued by hepatic expression of a nuclear isoform of TCF7L2 at the physiological level. Collectively, these data suggest a crucial role of TCF7L2 in hepatic glucose metabolism; reduced hepatic expression of nuclear isoforms of this factor might be a critical instigator of hyperglycemia in type 2 diabetes.

## Author Summary

Previous genome-wide association studies revealed that TCF7L2 is a strong candidate for a type 2 diabetes gene. However, the direct involvement of TCF7L2 on hepatic glucose metabolism has been elusive to date. Here we show that TCF7L2 is critical in mediating transcriptional control of hepatic glucose production. We found that hepatic expression of nuclear isoforms of TCF7L2 was reduced in mouse models of insulin resistance. Acute depletion of TCF7L2 in the liver promoted glucose intolerance and up-regulation of gluconeogenic genes, while ectopic expression of TCF7L2 in DIO mice improved glucose tolerance. TCF7L2 was shown to bind to the gluconeogenic promoters, thereby interfering with the promoter occupancies of both CREB/CRTC2 and FoxO1 on their cognate sites. Furthermore, TCF7L2 haploinsufficiency promoted higher glucose levels with impaired glucose tolerance and increased hepatic glucose production in mice, and adenovirus-mediated TCF7L2 expression in the liver reversed the phenotype. We propose that TCF7L2 is a critical player in regulating glucose homeostasis in mammals by modulating hepatic glucose production.

## Introduction

Dysregulation of hepatic glucose metabolism is a major predicament for the development of type 2 diabetes. During insulin resistant conditions, physiological activation of Akt-dependent pathway under feeding is impaired, which results in the failure to suppress hepatic glucose production in part via prolonged transcriptional activation of gluconeogenesis [1], [2], [3], [4]. Hepatic gluconeogenic gene expression is mainly controlled by two major transcriptional machineries, namely cAMP response element binding protein (CREB) Regulated Transcription Activator 2 (CRTC2, also known as TORC2) – CREB and Peroxisome Proliferation Activating Receptor Co-activator 1 alpha (PGC-1α) – FoxO1. Under fasting conditions, cAMP-dependent protein kinase (PKA) is critical in activating both machineries. PKA-dependent phosphorylation of CREB at Serine 133 promotes the recruitment of CREB binding protein (CBP)/p300 [5], [6], [7], [8], [9], [10]. Furthermore, PKA-dependent inhibition of AMP activated protein kinase (AMPK) and its related kinases (AMPKRK) results in the dephosphorylation and nuclear localization of CRTC2, promoting active complex formation of CRTC2-CREB-CBP/p300 on the promoters of gluconeogenic genes such as phosphoenol pyruvate carboxykinase (PEPCK) and glucose 6 phosphatase catalytic subunit (G6Pase) [11], [12], [13], [14], [15]. Similarly, AMPK/AMPKRK-dependent signal activates FoxO1-driven transcription by increasing nuclear retention of this factor via a HDAC-dependent manner [16]. PGC-1α itself is transcriptionally activated by CRTC2-CREB-CBP/p300, showing that PGC-1α-FoxO1 pathway is also under the control of the cAMP-dependent mechanism [17], [18]. The role of individual contribution of each factor, however, is currently under the debate. Recent paper by Lu et al. [19] showed the data suggesting that insulin could regulate hepatic gluconeogenic gene expression via FoxO1-independent manner, contesting the current model regarding the critical role of this factor as a regulatory target of insulin signaling pathways in the liver. Similarly, two groups reported the contrasting results using the independent lines of knockout mice for CRTC2 [20], [21]. These data collectively suggest that disruption of single transcriptional machinery might not be enough to affect hepatic glucose metabolism *in vivo*, and the transcriptional circuits are indeed tightly interwoven with each other for the fine tuning of glucose homeostasis.

First identified as a member of the T-cell factor (TCF) family possessing HMG-box-containing DNA-binding domain, TCF7L2 (also known as TCF4) has been known as a nuclear effector of Wnt/β-catenin pathway [22], [23], [24], [25]. Activation of Wnt signaling promotes accumulation and nuclear entry of β-catenin, enabling an association between this factor and TCF7L2 to promote target gene expression. Wnt/β-catenin signaling plays a crucial role in many developmental processes as well as in some adult mammalian tissues that are active in self-renewing processes such as proliferating crypt precursors and differentiated villus cells in the intestinal epithelium, epidermal stem cells in the hair follicle, hematopoietic stem cells, osteoblasts, and several types of cancer cells (reviewed in [26], [27]). Recent evidences also indicated a role of this pathway in type 2 diabetes. Extensive genome-wide association (GWA) studies revealed that TCF7L2 is a strong candidate for a type 2 diabetes gene, and several studies indicated that the presence of certain common single nucleotide polymorphisms (SNPs) in this gene might increase the incidence of this disease in human [28], [29], [30], [31], [32], [33], [34], [35]. Indeed, incretin hormone GLP-1 is induced by TCF7L2 in the intestinal endocrine L cells, and GLP-1-dependent pancreatic beta cell proliferation and insulin secretion also require TCF7L2, suggesting that alteration in its expression in certain target tissues might display glucose phenotypes in affected individuals [36], [37]. The functional role of TCF7L2 in hepatic glucose metabolism, however, has not been clearly stated to date.

Here we propose that TCF7L2 is critical in mediating transcriptional control of hepatic glucose production. We found that hepatic expression of medium and short isoforms of TCF7L2 was specifically reduced in mouse models of insulin resistance. Acute depletion of TCF7L2 in the liver resulted in higher blood glucose levels that were associated with increased glucose intolerance and up-regulation of gluconeogenic genes, while ectopic expression of nuclear TCF7L2 in C57BL/6 mice with diet-induced obesity (DIO) improved glucose tolerance. TCF7L2 was shown to bind to the promoters of PEPCK and G6Pase, thereby interfering with the association of both CRTC2 and FoxO1 on their cognate recognition sites on the chromatin. Furthermore, mice with global haploinsufficiency of TCF7L2 exhibited higher glucose levels and impaired glucose tolerance compared with the littermate control, and adenovirus-mediated two-fold expression of TCF7L2 almost completely reversed the phenotype. Taken together, we suggest that TCF7L2 would be a critical player in regulating glycemia in mammals by modulating hepatic gluconeogenic gene expression.

## Results

### Acute depletion of TCF7L2 results in increased expression of gluconeogenic genes in the liver

**Figure 1:**
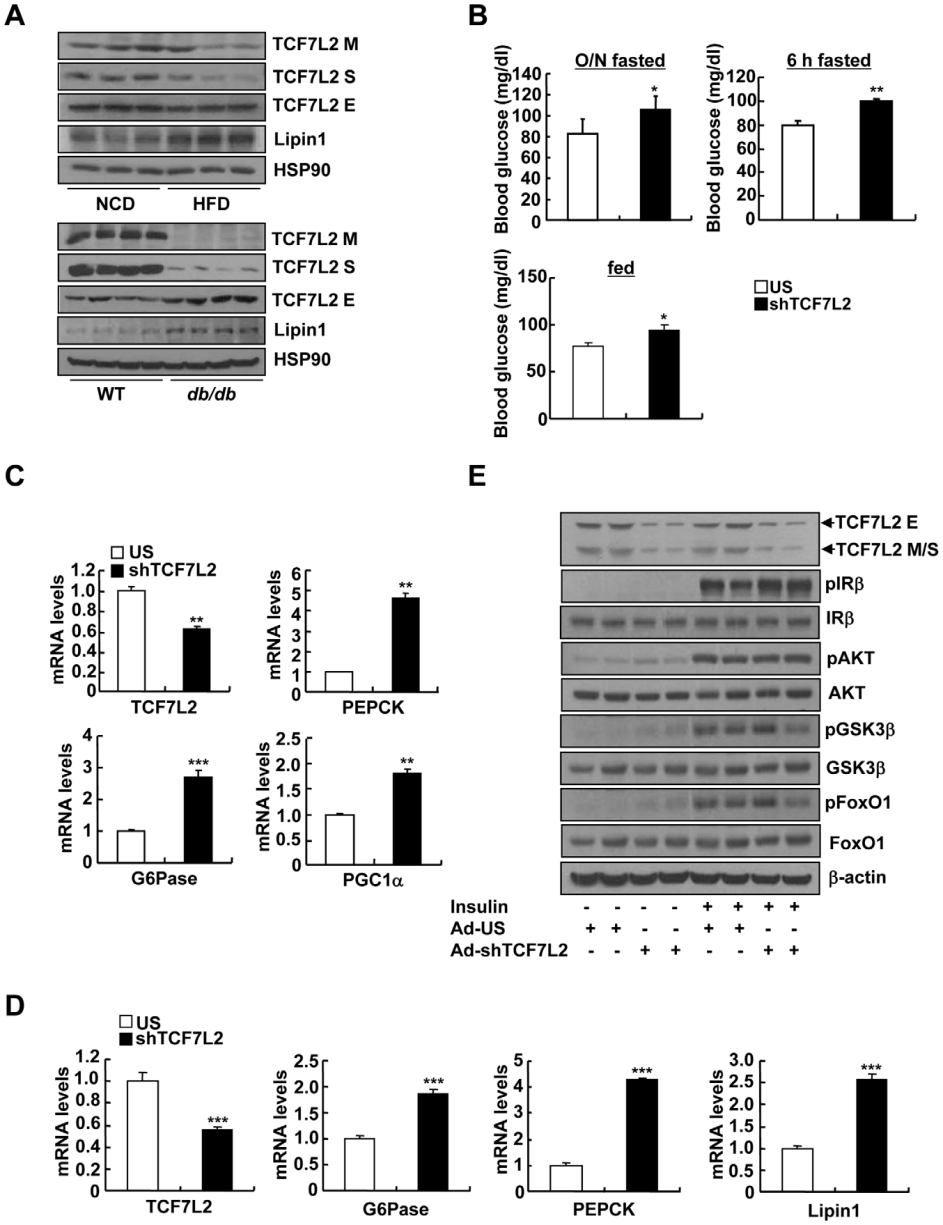
Knockdown of TCF7L2 promotes elevations in blood glucose levels in C57BL/6 mice. A) Western blot analysis showing protein expression levels of TCF7L2 M, TCF7L2 S, and TCF7L2 E in livers of high-fat diet-fed or normal chow diet-fed (top), and *db/db* or C57BL/6 mice (bottom). B) 16 h fasting glucose levels (top, left), 6 h fasting glucose levels (top, right), or feeding glucose levels (bottom) from 8-week-old C57BL/6 male mice that were infected with Ad-US (*n* = 7) or Ad-shTCF7L2 (*n* = 6). C) Q-PCR analysis showing effects of Ad-US (*n* = 3) or Ad-shTCF7L2 (*n* = 4) on hepatic expression of PEPCK, G6Pase, and PGC1α in C57BL/6 mice fasted for 6 h. D) Q-PCR analysis showing effects of Ad-US (*n* = 4) or Ad-shTCF7L2 (*n* = 4) on hepatic expression of G6Pase, PEPCK, and Lipin1 in C57BL/6 mice under feeding conditions. E) Western blot analysis showing effects of Ad-shTCF7L2 on insulin signaling pathway in mice. C57BL/6 mice infected with either Ad-US or Ad-shTCF7L2 for 5 days were fasted for 6 h, and then were given a bolus of insulin or saline for 10 min before being sacrificed. Data in B) represent mean ± SEM, and data in C) and D) represent mean ± SD (*;*P*<0.05, **;*P*<0.005, ***;*P*<0.0005, t-test).

Although TCF7L2 has been regarded as one of the major candidate genes for inducing type 2 diabetes, the exact role for this factor in hepatic glucose metabolism has not been well documented. To investigate the potential role for TCF7L2 in the liver, we firstly measured the expression level of TCF7L2 in livers of mice with various dietary conditions. Interestingly, overnight fasting or high-fat diet invoked reduced protein levels of only medium and short isoforms of TCF7L2 (designated as M and S, respectively) compared with control, while no change was shown in the expression levels of long isoforms (designated as E) (Figure 1A and Figure S1A). Furthermore, decreased expression of medium and short isoforms was also pronounced in the livers of *db/db* mice compared with control, suggesting that hepatic insulin resistance might be correlated with the disappearance of smaller isoforms of TCF7L2 in the liver (Figure 1A). While both medium and short isoforms of TCF7L2 primarily resided in the nucleus, a majority of long isoforms were found in the cytoplasm (Figure S1B). Since the expression of TCF7L2 was up-regulated under feeding, we wanted to further delineate the potential signaling cascades that are involved in this phenomenon. Unlike our expectations, treatment of insulin alone did not provoke changes in expression of TCF7L2 in primary hepatocytes, showing only a slight induction of both mRNA and protein expression with 24 h-treatment (Figure S1C). Addition of forskolin, a cAMP agonist, resulted in the reduction of TCF7L2 expression both at the mRNA and protein levels, suggesting that the disappearance of glucagon/cAMP signaling pathway, rather than the activation of insulin signaling pathway under feeding conditions, might be involved in the regulation of TCF7L2 expression (Figure S1D).

To explore the causal role of TCF7L2 in hepatic glucose metabolism, we generated an adenovirus expressing shRNA for TCF7L2 (Ad-TCF7L2 shRNA) and injected into the tail vein of C57BL/6 mice. Knockdown of all isoforms of hepatic TCF7L2 resulted in higher glucose levels with a slight increase in plasma insulin levels under both fasting and feeding conditions. No changes were observed in body weight, plasma and liver triacylglycerol (TG) levels, as well as plasma non-esterified fatty acid (NEFA) levels between mice injected with either Ad-TCF7L2 shRNA or control Ad-US virus, excluding a potential non-specific effect (Figure 1B, and Figure S2A-S2D). Glucose intolerance was observed in TCF7L2-knockdown mice compared with control, suggesting that insulin signaling might be perturbed with acute depletion of TCF7L2 in mice (Figure S2E). Excluding a change in insulin signaling, the rate of insulin-dependent clearance of blood glucose was not different between two groups as evidenced by the insulin tolerance test (Figure S2F). Since TCF7L2 is a transcription factor that could potentially affect glucose metabolism at the transcriptional level, we attempted to measure the relative expression levels of genes involved in glucose and glycogen metabolism between two groups (control vs. TCF7L2-knockdown) by PCR array analysis. Interestingly, expression levels of genes that are involved in gluconeogenesis were increased upon TCF7L2 knockdown (PEPCK, G6Pase, Fructose 1, 6-bisphosphatase 1 (Fbp1), and Fructose 1, 6-bisphosphatase 2 (Fbp2)) in mouse liver (Table 1). As well, genes encoding Fumarase (FH1) and Malate dehydrogenase (Mdh1b), two enzymes that are critical in providing malate for gluconeogenesis from the mitochondrial TCA cycle, and pyruvate dehydrogenase kinase 4 (PDK4), which functions to reduce the formation of acetyl CoA and block the TCA cycle, were also significantly induced with depletion of TCF7L2 in the liver. Indeed, we were able to confirm the significant induction in the expression of gluconeogenic genes in the livers of TCF7L2-knockdown mice compared with that of control by Q-PCR, suggesting that hepatic gluconeogenic potential is specifically enhanced upon depletion of TCF7L2 in the mouse liver (Figure 1C and 1D). As hinted by the result from the insulin tolerance test, knockdown of TCF7L2 did not alter the phosphorylation status of key enzymes in the hepatic insulin signaling (Figure 1E and Figure S3A), suggesting that the changes in the expression level of TCF7L2 *per se* might not be directly linked to the fluctuation in the insulin signaling pathway in the liver. Similar results were also obtained in primary hepatocytes using Ad-shTCF7L2, further supporting the direct role of TCF7L2 in the regulation of hepatic gluconeogenic gene expression (Figure S3B–S3E).

**Table 1:** Results of Glucose and Glycogen metabolism PCR Array (US, *n* = 4; shTCF7L2, *n* = 4).

	Symbol	shTCF7L2/US (Fold induction)	*p*-value		Symbol	shTCF7L2/US (Fold induction)	*p*-value
**Gluconeogenesis**	Pck1	1.614	*p*<.05	**Pentose phosphate pathway**	Prps1l1	1.258	*p*<.05
	G6pc	1.47	*p*<.05		Prps1	1.134	N.S
	G6pc3	1.228	N.S		H6pd	1.076	N.S
	Fbp1	1.223	*p*<.05		Rbks	1.073	N.S
	Fbp2	1.181	*p*<.05		Rpe	1.069	N.S
	Pck2	1.099	N.S		G6pdx	1.011	N.S
	Pcx	1.043	N.S		Prps2	1.001	N.S
**Glycolysis**	Gpi1	1.44	N.S		Taldo1	0.943	N.S
	Aldoa	1.198	N.S		Tkt	0.889	N.S
	Pgk2	1.19	N.S	**TCA cycle**	Fh1	1.62	*p*<.005
	Aldob	1.178	*p*<.05		Mdh1b	1.262	*p*<.05
	Pgm2	1.178	N.S		Idh3a	1.19	N.S
	Eno3	1.157	N.S		Mdh2	1.178	N.S
	Galm	1.099	N.S		Aco1	1.13	N.S
	Eno1	1.087	N.S		Aco2	1.099	N.S
	Hk2	1.084	N.S		Cs	1.084	N.S
	Aldoc	1.063	N.S		Idh3g	1.08	N.S
	Pklr	1.05	N.S		Dlat	1.076	N.S
	Pgm3	1.032	N.S		Dlst	1.058	N.S
	Eno2	1.028	N.S		Idh3b	1.058	N.S
	Pgk1	1.022	N.S		Acly	1.054	N.S
	Pfkl	1.022	N.S		Sdha	1.043	N.S
	Hk3	1.015	N.S		Mdh1	1.029	N.S
	Pgm1	1.001	N.S		Idh2	1.025	N.S
	Gapdhs	0.967	N.S		Sucla2	1.011	N.S
	Bpgm	0.967	N.S		Suclg2	1.004	N.S
	Tpi1	0.957	N.S		Pdha1	0.98	N.S
	Pgam2	0.953	N.S		Idh1	0.973	N.S
	Gck	0.796	N.S		Ogdh	0.97	N.S
**Regulation of glucose metabolism**	Pdk4	1.7	*p*<.005		Suclg1	0.953	N.S
	Pdk3	1.126	N.S		Sdhb	0.934	N.S
	4833426J09Rik	0.987	N.S		Dld	0.934	N.S
	Pdk1	0.96	N.S		Sdhc	0.93	N.S
	Pdk2	0.905	N.S		Sdhd	0.902	N.S
**Glycogen synthesis**	Gbe1	1.114	N.S		Pdhb	0.874	N.S
**Glycogen synthesis (cont.)**	Gys2	1.114	N.S	**Regulation of glycogen metabolism**	Phka1	1.207	N.S
	Gys1	1.054	N.S		Gsk3a	1.202	*p*<.05
	Ugp2	0.973	N.S		Gsk3b	1.198	N.S
**Glycogen degradation**	Agl	1.142	N.S		Phkb	1.162	*p*<.05
	Pygm	1.054	N.S		Phkg1	1.058	N.S
	Pygl	0.994	N.S		Phkg2	0.886	N.S

### TCF7L2 relieves high-fat diet-induced hyperglycemic phenotypes in mice

**Figure 2:**
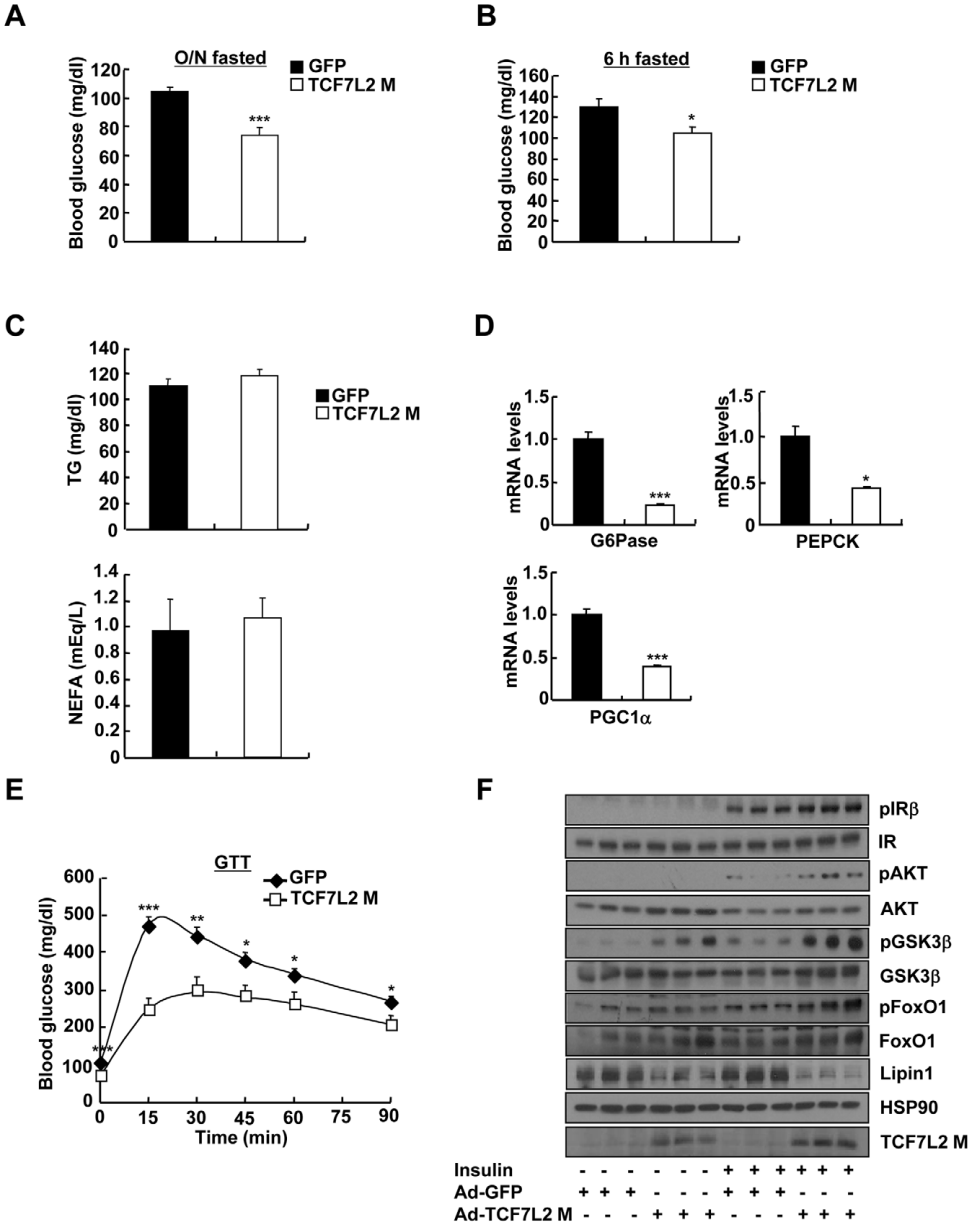
Ectopic expression of TCF7L2 in the liver alleviates impaired glucose metabolism in high-fat diet-fed mice. A) 16 h fasting glucose levels from high-fat diet-fed 14-week-old C57BL/6 male mice that were infected with Ad-GFP (*n* = 10) or Ad-TCF7L2 M adenovirus (*n* = 7). B) 6 h fasting glucose levels from high-fat diet-fed 14-week-old C57BL/6 male mice that were infected with Ad-GFP (*n* = 10) or Ad-TCF7L2 M adenovirus (*n* = 7). C) Plasma TG and NEFA levels from high-fat diet-fed 14-week-old C57BL/6 male mice that were infected with Ad-GFP (*n* = 10) or Ad-TCF7L2 M adenovirus (*n* = 7). D) Q-PCR analysis showing effects of Ad-GFP (*n* = 4) or Ad-TCF7L2 M (*n* = 4) on hepatic expression of gluconeogenic genes. E) Glucose tolerance test showing effects of TCF7L2 expression on glucose homeostasis (*n* = 10 for Ad-GFP, and *n* = 7 for Ad-TCF7L2 M). F) Western blot analysis showing effects of Ad-TCF7L2 on insulin signaling pathway in mice. High-fat diet-fed C57BL/6 mice infected with either Ad-GFP or Ad-TCF7L2 for 5 days were fasted for 6 h, and then were given a bolus of insulin or saline for 10 min before being sacrificed. Data in A–C) and E) represent mean ± SEM, and data in D) represent mean ± SD (*;*P*<0.05, **;*P*<0.005, ***;*P*<0.0005, t-test).

Depletion of hepatic TCF7L2 promoted higher glucose levels, suggesting that reduced expression of certain isoforms of TCF7L2 under insulin resistance might be in part responsible for the hyperglycemia in that setting. To test this hypothesis, we generated adenoviruses expressing various isoforms of TCF7L2 (Ad-TCF7L2 M, Ad-TCF7L2 S, and Ad-TCF7L2 E), and tested their effects on expression of gluconeogenic genes in primary hepatocytes. TCF7L2 M and S, nuclear isoforms that displayed reduced expression in livers of insulin resistant mice, were more effective in inhibiting expression of gluconeogenic genes than the cytosolic TCF7L2 E, suggesting that the effect of TCF7L2 might occur largely in the nucleus (Figure S4A). We thus chose to utilize adenovirus expressing TCF7L2 M, a widely used isoform for various studies, for our *in vivo* experiments. Indeed, adenovirus-mediated expression of TCF7L2 M diminished fasting blood glucose levels without changes in body weight in DIO mice (Figure 2A, 2B, and Figure S4B). No changes were observed in plasma TG and NEFA levels between mice injected with either Ad-TCF7L2 M or control Ad-GFP (Figure 2C). Neither insulin tolerance nor plasma insulin levels was changed with expression of TCF7L2, suggesting that global insulin signaling might not be affected by Ad-TCF7L2 M infection (Figure S4C and S4D). Mice with Ad-TCF7L2 M displayed reduction in gluconeogenic gene expression, showing that indeed TCF7L2 could be linked to the regulation of glucose homeostasis by inhibiting expression of gluconeogenic genes (Figure 2D). On the other hand, glucose tolerance was significantly improved in mice expressing TCF7L2 compared with control, and hepatic insulin signaling appeared to be slightly improved by TCF7L2 overexpression in the liver as evidenced by increased tyrosine phosphorylation of IRβ and serine phosphorylation of AKT, GSK3β, and FoxO1, presumably due to the secondary effect that was associated with improved glycemia in DIO mice (Figure 2E and 2F). Indeed, we did not observe changes in hepatic insulin signaling with Ad-TCF7L2 infection in lean mice, suggesting that TCF7L2 might not directly regulate insulin signaling in the physiological context (data not shown).

**Figure 3:**
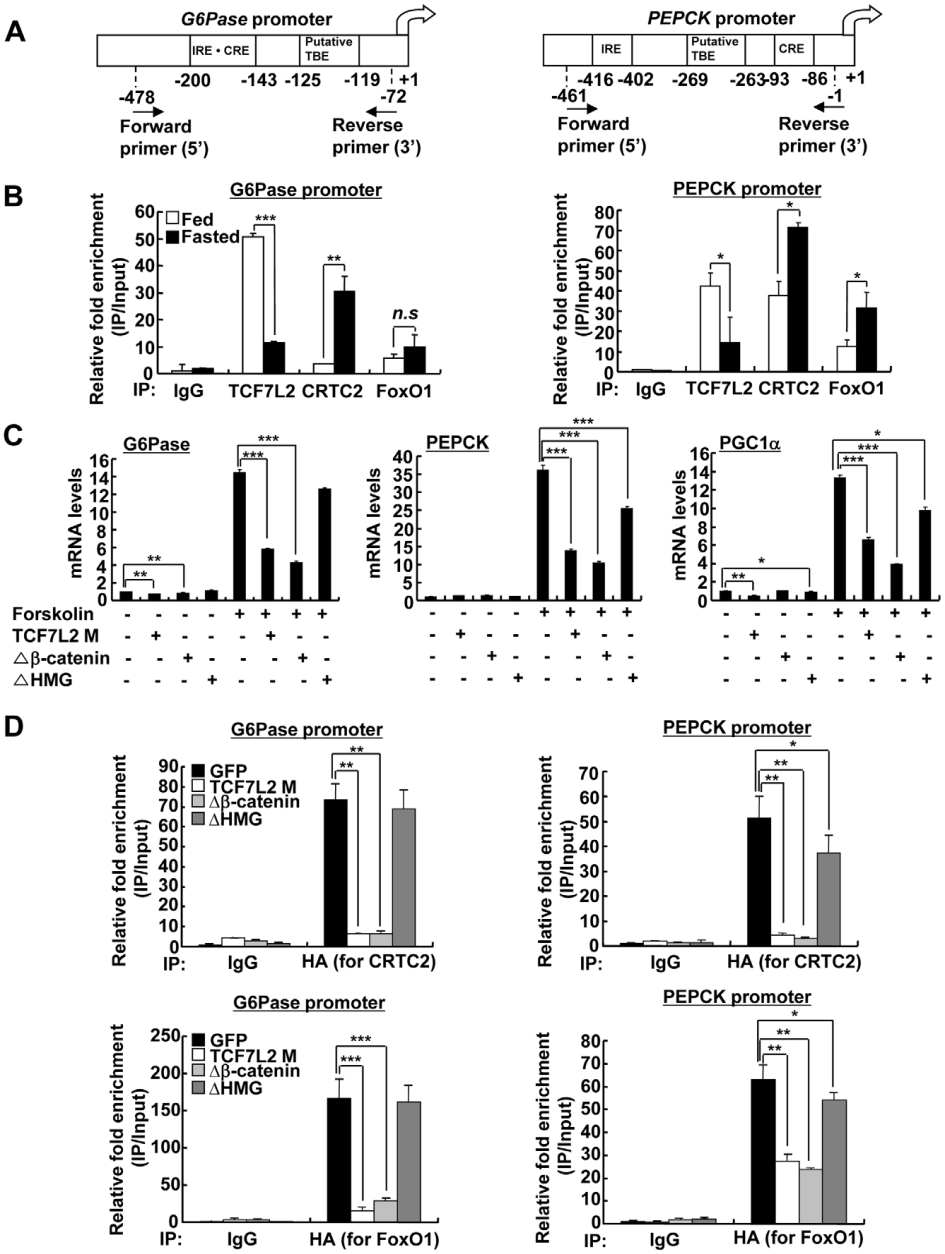
Ectopic expression of TCF7L2 inhibits gluconeogenesis at the transcription level. A) Schematic representation of G6Pase (right) and PEPCK promoters (left). IRE: Insulin response element, CRE: cAMP response element, TBE: TCF binding element. B) Chromatin immunoprecipitation assay showing occupancy of TCF7L2 or CRTC2/FoxO1 over G6Pase and PEPCK promoters under feeding and fasting conditions. Representative data from at least three independent experiments are shown. C) Q-PCR analysis showing effects of TCF7L2 wild type and mutants on expression levels of gluconeogenic genes in mouse primary hepatocytes (*n* = 3 for each group). ΔHMG: TCF7L2 containing mutations in DNA binding motif, Δβ-catenin: TCF7L2 containing mutations in the beta-catenin binding motif. Representative data from at least three independent experiments are shown. D) Chromatin immunoprecipitation assay showing effects of TCF7L2 wild type and mutants on CRTC2 or FoxO1 occupancy over G6Pase and PEPCK promoters in mouse primary hepatocytes. Occupancy of CRTC2 (top) or FoxO1 (bottom) over G6Pase and PEPCK promoters was shown. Data are shown as the relative enrichment of IP/input ratios of each antibody over that of IgG control. Representative data from at least three independent experiments are shown. Data in B–D) represent mean ± SD (*;*P*<0.05, **;*P*<0.005, ***;*P*<0.0005, t-test).

**Figure 4:**
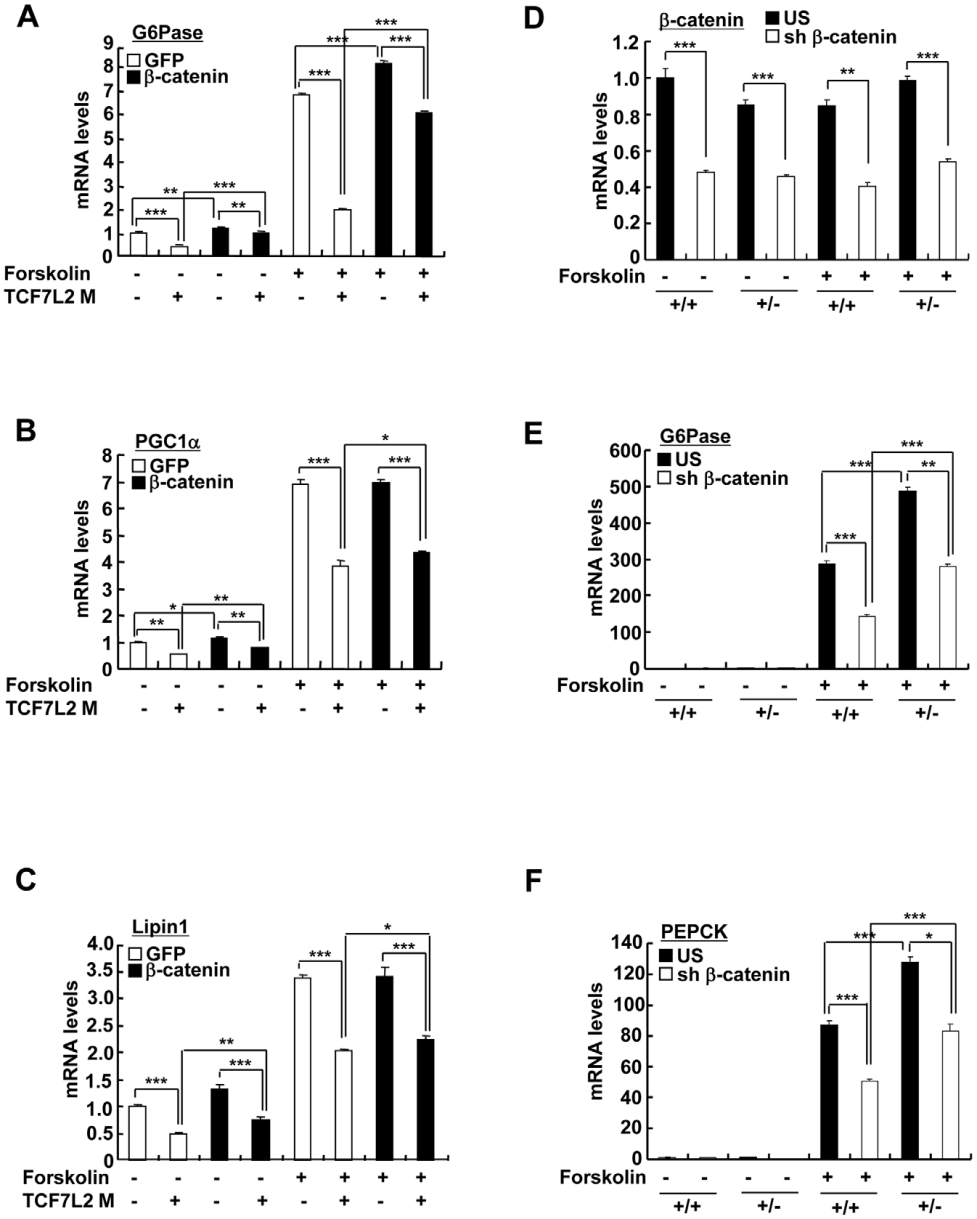
TCF7L2 does not require β-catenin for the regulation of hepatic glucose metabolism. (A–C) Q-PCR analysis showing effects of β-catenin expression on TCF7L2-dependent regulation of gluconeogenic genes in primary hepatocytes (*n* = 3 for each group). Representative data from at least three independent experiments are shown. D–F) Q-PCR analysis showing effects of β-catenin knockdown on TCF7L2-dependent regulation of gluconeogenic gene expression in primary hepatocytes from either *TCF7L2* +/+ (+/+) or *TCF7L2* +/− mice (+/−) (*n* = 3 for each group). Representative data from at least three independent experiments are shown. Data in A–F) represent mean ± SD (*;*P*<0.05, **;*P*<0.005, ***;*P*<0.0005, t-test).

Next, we wanted to verify whether TCF7L2 is directly involved in the transcriptional control of gluconeogenic genes. Indeed, we were able to recapitulate the inhibitory effect of TCF7L2 on glucose production in primary hepatocytes without changes in insulin signaling pathways, ruling out the potential involvement of other organs or cell types upon adenoviral delivery *in vivo* (Figure S4E and S4F). Furthermore, reporter assay revealed that both PEPCK and G6Pase promoter activities were inhibited by ectopic expression of TCF7L2 (Figure S4G), providing an evidence for the involvement of direct binding of TCF7L2 on the promoters of gluconeogenic genes. Careful investigation of promoter sequences revealed the presence of putative TCF binding elements (TBEs) that is adjacent to the CREB/CRTC2 binding site (cAMP response element, CRE) and the FoxO1 binding site (insulin response element, IRE) on both PEPCK and G6Pase promoters (Figure 3A). Consistent with the proposed role of TCF7L2 in inhibiting gluconeogenic gene expression under feeding conditions, we observed the reciprocal and mutually exclusive binding of TCF7L2 or CRTC2/FoxO1 onto the promoters of gluconeogenic genes under fasting and feeding conditions. By chromatin immunoprecipitation (ChIP) assay, we detected an increase in occupancy of TCF7L2 and a decrease in occupancy of CRTC2/FoxO1 over PEPCK or G6Pase promoter under feeding, while increased binding of CRTC2/FoxO1 and decreased binding of TCF7L2 onto these promoters were evident under fasting conditions in mouse liver (Figure 3B). We speculated that the reduced expression of TCF7L2 under fasting conditions might in part contribute to the increased occupancy of CREB/CRTC2 or FoxO1 over gluconeogenic promoters. Mutations in TBE site blunted inhibitory effects of TCF7L2 on activity of gluconeogenic promoters in cultured cells (Figure S5A). To further provide the evidence for the importance of the ability of TCF7L2 to bind DNA in inhibiting gluconeogenic gene expression, we generated two types of mutants; TCF7L2 Δβ-catenin mutant, which contains an intact DNA binding motif but lacks a β-catenin interaction domain, and TCF7L2 ΔHMG mutant, which retains a β-catenin interaction domain but lacks a DNA binding motif (Figure S5B and S5C). In line with this result, mutations on DNA binding motif (ΔHMG), but not on the beta-catenin binding motif Δβ-catenin), completely impaired the ability of TCF7L2 to inhibit gluconeogenic gene expression (Figure 3C). These data suggest that while binding to β-catenin is dispensable, the ability to bind to the gluconeogenic promoters is essential for the inhibitory function of TCF7L2. ChIP assay also revealed that ectopic expression of TCF7L2 WT or Δβ-catenin, but not of ΔHMG, inhibited the occupancy of CRTC2 or FoxO1 on the cognate binding sites of the gluconeogenic promoters (Figure 3D). Instead, increased binding of TCF7L2 to the adjacent putative TCF binding element (TBE) on the chromatin was observed (Figure S5D), suggesting that TCF7L2 would inhibit transcription of gluconeogenic genes by binding to the promoter and inhibiting the formation of active transcription factor complex in hepatocytes. To further assess the potential involvement of β-catenin, a known co-activator for TCF7L2, in the TCF7L2-dependent inhibition of gluconeogenic gene expression, we generated adenovirus for β-catenin expression, and tested in primary hepatocytes. We found that overexpression of β-catenin did not promote the inhibitory effect of TCF7L2 on the expression of G6Pase, PGC1α, or Lipin1, known targets for FoxO1 and CREB/CRTC2 (Figure 4A–4C). Furthermore, knockdown of β-catenin rather reduced the forskolin-induced expression of G6Pase and PEPCK in the absence of TCF7L2, suggesting that β-catenin and TCF7L2 did not function in concert at least for the regulation of gluconeogenic genes in the liver (Figure 4D–4F).

### Haploinsufficiency of TCF7L2 promotes increased hepatic glucose production

**Figure 5:**
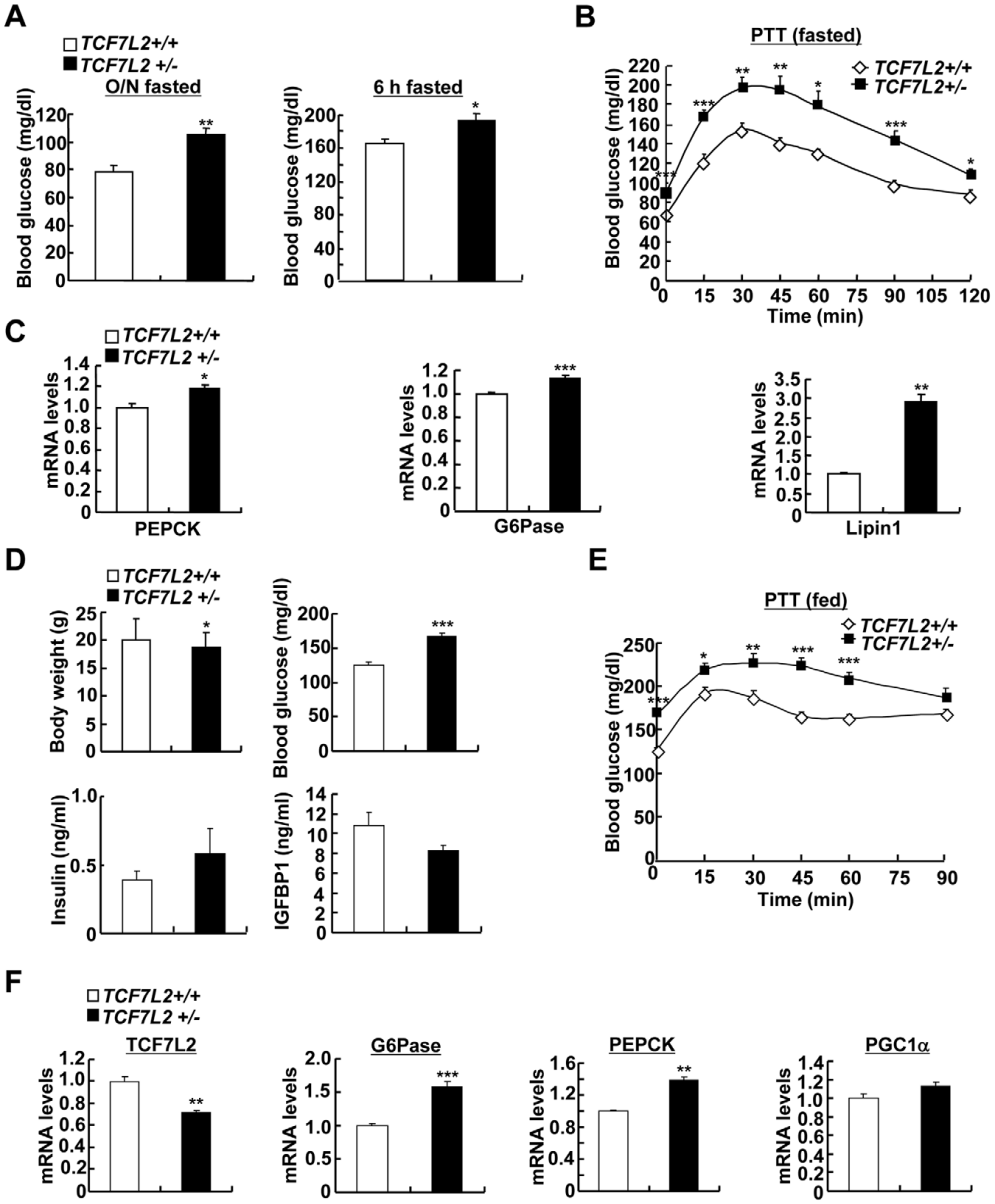
Chronic depletion of TCF7L2 promotes increased glucose production from the liver. A) Effects of haploinsufficiency of TCF7L2 on glucose metabolism. 16 h fasting glucose levels (left) or 6 h fasting glucose levels (right) from 8-week-old *TCF7L2 +/+* (*n* = 10) and *TCF7L2 +/−* (*n* = 10) male mice under the normal chow diet were shown. B) Pyruvate tolerance test showing effects of chronic depletion of TCF7L2 on glucose homeostasis under 16 h fasting conditions (*n* = 10 for *TCF7L2* +/+ mice, and *n* = 10 for *TCF7L2* +/− mice). C) Q-PCR analysis showing expression levels of gluconeogenic genes in livers of *TCF7L2 +/+* and *TCF7L2 +/−* mice fasted for 6 h (*n* = 5 for *TCF7L2* +/+ mice, and *n* = 5 for *TCF7L2* +/− mice). D) Effects of haploinsufficiency of TCF7L2 on body weight, blood glucose, serum insulin, and serum IGFBP1 levels under feeding conditions (*n* = 8 for *TCF7L2* +/+ mice, and *n* = 8 for *TCF7L2* +/− mice). E) Pyruvate tolerance test showing effects of chronic depletion of TCF7L2 on glucose homeostasis under feeding conditions (*n* = 8 for *TCF7L2* +/+ mice, and *n* = 8 for *TCF7L2* +/− mice). F) Q-PCR analysis showing expression levels of gluconeogenic genes in livers of *TCF7L2 +/+* and *TCF7L2 +/−* mice under feeding conditions (*n* = 7 for *TCF7L2* +/+ mice, and *n* = 7 for *TCF7L2* +/− mice). Data in A), B), D), and E) represent mean ± SEM, and data in C) and F) represent mean ± SD (*;*P*<0.05, **;*P*<0.005, ***;*P*<0.0005, t-test).

To ascertain whether chronic depletion of TCF7L2 in the liver might play a causal role in the promotion of hyperglycemia, we obtained knockout mice for TCF7L2 gene in C57BL/6 background from Sanger Institute. As in the case of previously generated lines, we were not able to obtain viable TCF7L2 homozygous knockout mice. Thus, we bred heterozygous null mice (*TCF7L2* +/−) to produce TCF7L2 heterozygous null mice (*TCF7L2 +/−*) and their littermates (*TCF7L2 +/+*) for the subsequent study (Figure S6A). In accordance with the effect of the acute depletion of TCF7L2 in mice, *TCF7L2 +/−* mice displayed higher blood glucose levels with no significant changes in plasma insulin levels compared with their littermates under fasting (Figure 5A, Figure S6B and S6C). In addition, *TCF7L2+/−* mice also displayed pyruvate intolerance that was accompanied with increased hepatic expression of gluconeogenic genes, suggesting that chronic depletion of TCF7L2 might promote increased glucose production from the liver (Figure 5B and 5C). Similar results on blood glucose levels, plasma metabolites levels, and gluconeogenic gene expression were also obtained using *TCF7L2* +/− mice under feeding conditions (Figure 5D and 5E). Glucose intolerance was also apparent in *TCF7L2* +/− mice compared with control (Figure S6D, top). Excluding a potential involvement of pancreatic beta cells, we were not able to observe a difference in glucose-induced insulin levels between two groups of mice (Figure S6D, bottom). Hepatic glycogen levels were reduced in *TCF7L2* +/− mice compared with control, suggesting that glycogen metabolism might be affected by haploinsufficiency of TCF7L2 in mice (Figure S6E). To evaluate the potential changes in whole body insulin sensitivity, we performed hyperinsulinemic-euglycemic clamp studies. Compared with the control, we observed increased glucose production from *TCF7L2+/−* mice, although the statistical significance was only observed at the basal period (Figure S6F). However, no specific changes were observed in whole body glucose metabolism during the clamp period between *TCF7L2 +/+* mice and *TCF7L2 −/−* mice, even in the presence of mild reduction in body weight and muscle mass upon TCF7L2 haploinsufficiency, suggesting that haploinsufficiency of TCF7L2 might not invoke changes in peripheral insulin signaling pathway at least under the normal chow diet conditions (Figure S6F and S6G). In accordance with this phenomenon, we were not able to observe differences in phosphorylation status of key insulin signaling enzymes in the liver, pancreas, adipose tissues, or skeletal muscle between wild type and *TCF7L2*+/− mice (Figure S7A–S7D).

**Figure 6:**
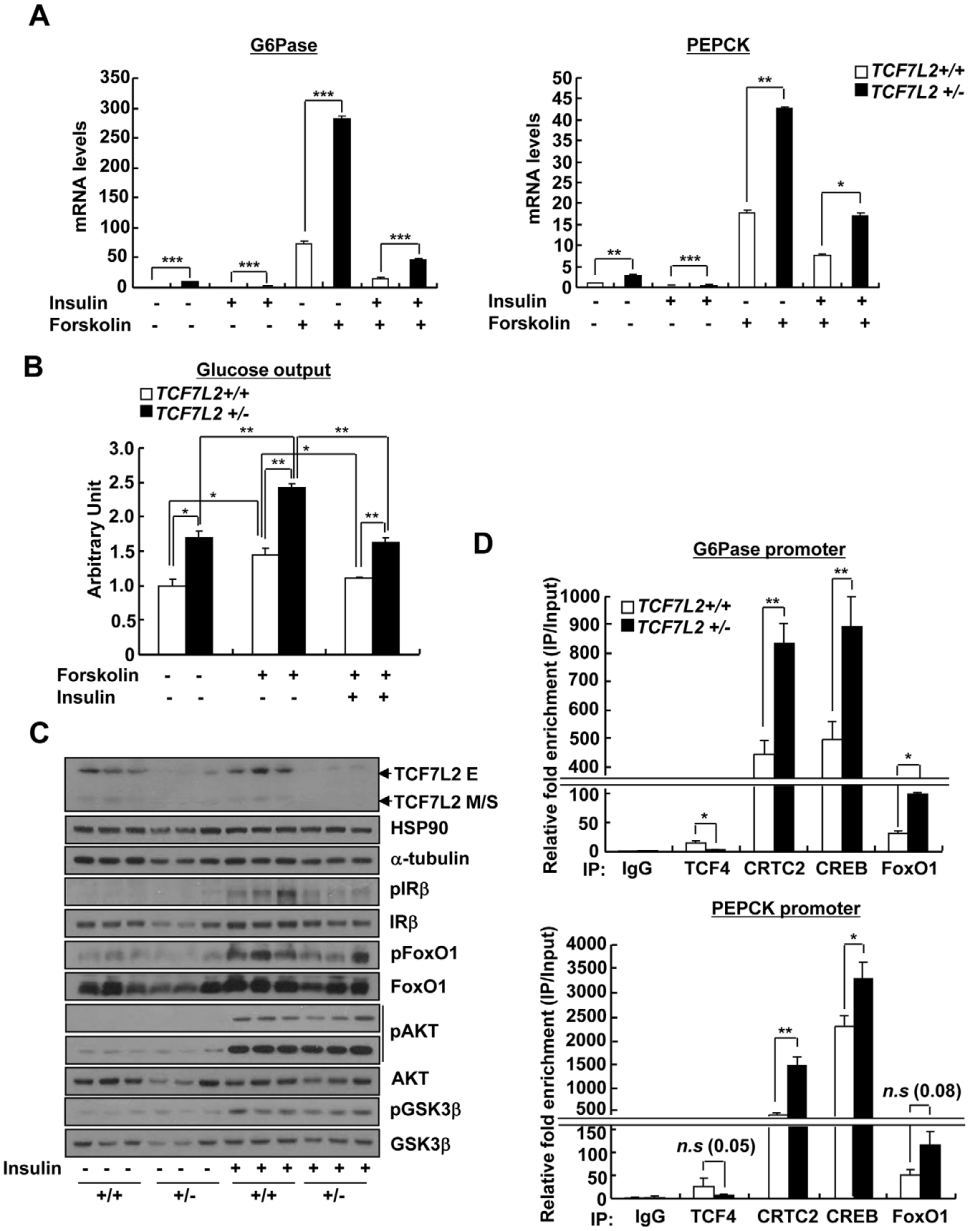
Chronic depletion of TCF7L2 promotes increased glucose production and gluconeogenic gene expression in hepatocytes. A) Q-PCR analysis showing effects of forskolin (10 µm, 2 h) and insulin (100 nM, 12 h) on expression levels of gluconeogenic genes in primary hepatocytes from *TCF7L2 +/+* and *TCF7L2 +/−* mice (*n* = 3 for each group). Representative data from at least three independent experiments are shown. B) Glucose output assay showing the effects of TCF7L2 levels on glucose production between primary hepatocytes from *TCF7L2 +/+* or *TCF7L2 +/−* mice was performed as described in Materials and Methods (*n* = 3 for each group). Representative data from at least three independent experiments are shown. C) Western blot analysis showing insulin signaling in primary hepatocytes from *TCF7L2 +/+* and *TCF7L2 +/− mice*. Cells were treated with 100 nM insulin for 15 min. Representative data from at least three independent experiments are shown. D) Chromatin immunoprecipitation experiments showing effects of TCF7L2 depletion on endogenous CREB, CRTC2, or FoxO1 occupancy over G6Pase and PEPCK promoters in primary hepatocytes from *TCF7L2 +/+* or *TCF7L2 +/−* mice. Antibodies against each protein were utilized to detect the association of endogenous transcription factors with the chromatin. Data are shown as the relative enrichment of IP/input ratios of each antibody over that of IgG control. Representative data from at least three independent experiments are shown. Data in A), and D) represent mean ± SD, and data in B) represent mean ± SEM (*;*P*<0.05, **;*P*<0.005, ***;*P*<0.0005, t-test).

To analyze the liver-specific effect of chronic depletion of TCF7L2, we prepared primary hepatocytes from either *TCF7L2+/−* mice or *TCF7L2+/+* mice. Chronic haploinsufficiency of TCF7L2 indeed displayed higher levels of gluconeogenic gene expression and increased glucose production in primary hepatocytes, without impairment of normal insulin signaling (Figure 6A–6C). Similar to the clamp studies *in vivo*, we were able to observe the increased glucose production from the *TCF7L2* +/− hepatocytes compared with control. Again, insulin was able to repress the forskolin-induced glucose production from hepatocytes of both genotypes, showing insulin signaling itself was not perturbed by haploinsufficiency of TCF7L2. Furthermore, increased occupancy of endogenous CREB, CRTC2, or FoxO1, with concomitant decrease in the occupancy of endogenous TCF7L2, on the gluconeogenic promoter was apparent in *TCF7L2+/−* hepatocytes compared with control cells (Figure 6D). These data once again suggest that binding of TCF7L2 and CRTC2/FoxO1 on the promoters of gluconeogenic genes might be mutually exclusive, and that the haploinsufficiency of hepatic TCF7L2 is indeed critical in promoting dysregulation of hepatic glucose production. To further ascertain that the effects of TCF7L2 on the hepatic gluconeogenic gene expression function by direct inhibition of CRTC2 and FoxO1 activities, we performed knockdown of both factors in primary hepatocytes from *TCF7L2* +/− mice. Increased mRNA levels of PEPCK and G6Pase by haploinsufficiency of TCF7L2 were indeed greatly normalized by knockdown of CRTC2 and FoxO1, showing that TCF7L2-dependent regulation of hepatic gluconeogenic gene expression directly modulated activities of these transcriptional machineries (Figure S8A and S8B).

### Ectopic expression of TCF7L2 restores euglycemia and glucose tolerance in TCF7L2 heterozygous knockout mice

**Figure 7:**
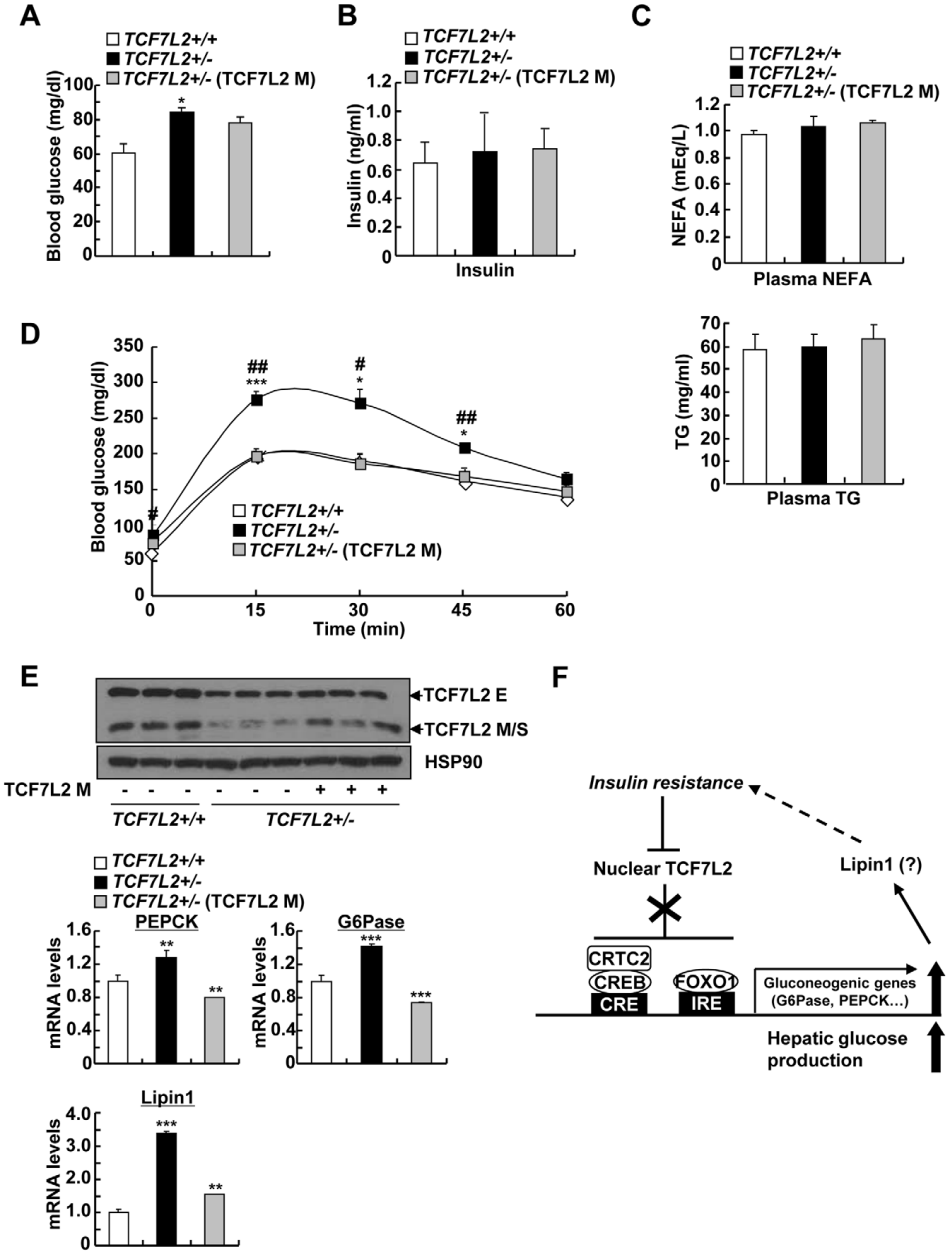
Mild ectopic expression of TCF7L2 M in the liver improves glycemic phenotypes in TCF7L2+/- mice. A–C) 8 week-old *TCF7L2* +/− mice were infected with Ad-GFP (*n* = 5) or Ad-TCF7L2 M adenovirus (*n* = 5), while their littermates (*TCF7L2* +/+ mice) were infected with Ad-GFP (*n* = 5) for 5 days. 16 h fasting glucose levels (A), 16 h fasting plasma insulin (B), as well as plasma NEFA (middle), and TG levels (bottom) (C) were shown. D) Glucose tolerance test showing effects of hepatic re-expression of TCF7L2 in TCF7L2 +/− mice on glucose homeostasis (*n* = 5 for each group) (*; *TCF7L2* +/+ (GFP) vs. *TCF7L2* +/− (GFP), #; *TCF7L2* +/− (GFP) vs. *TCF7L2* +/− (TCF7L2 M)). E) Western blot analysis (top) and Q-PCR analysis (bottom) showing effects of hepatic re-expression of TCF7L2 in TCF7L2 +/− mice on hepatic expression of gluconeogenic genes (*n* = 5 for each group). F) Schematic diagram showing the proposed mechanism for the regulation of TCF7L2 and subsequent gluconeogenic program in the liver. Hepatic insulin resistance promotes hyperglycemia by reducing expression of nuclear TCF7L2. Stimulation of CREB- and FoxO1-dependent transcriptional pathway may provide the additional link (e.g. Lipin1) for the further exacerbation of insulin resistance. Data in A–D) represent mean ± SEM, and data in E) represent mean ± SD (*;*P*<0.05, **;*P*<0.005, ***;*P*<0.0005, t-test).

To further support the hypothesis that impaired glucose metabolism in global haploinsufficiency of TCF7L2 in mice is largely due to the problems in the liver, we used adenovirus expressing TCF7L2 M to restore the expression of TCF7L2 specifically in the liver. We did not detect expression of TCF7L2 M expression in other insulin sensitive tissues such as pancreatic islet, skeletal muscle, or adipose tissues upon adenoviral infection (data not shown). Restoration of TCF7L2 expression in the liver of *TCF7L2 +/−* mice slightly reduced fasting glucose levels with reduction in expression levels for gluconeogenic genes that were largely comparable with those of wild type mice, without promoting changes in plasma insulin, NEFA, and TG levels (Figure 7A–7C). Glucose intolerance that was associated with global haploinsufficiency of TCF7L2 was almost completely abolished by hepatic expression of TCF7L2 (Figure 7D and 7E). These data collectively suggest that the glucose phenotype that is associated with *TCF7L2 +/−* mice might be in part due to the dysregulation of glucose metabolism in the liver.

## Discussion

Common SNPs of TCF7L2 such as rs7903146 and rs12255372 are associated with type 2 diabetes. Indeed, several studies indicated that patients carrying these SNPs might have the increased risk for the development of this disease [38], [39], [40], [41]. The observed SNPs, however, are localized in the intronic regions of TCF7L2 gene, and several attempts to correlate the presence of the intronic SNPs with changes in expression of this gene in various tissues such as adipose tissue, skeletal muscle, and pancreatic islets have been largely inconclusive [40], [42], [43], [44], [45]. Recent study provided the direct evidence against this hypothesis by showing no correlation between type 2 diabetes-associated SNPs and relative expression of this gene in adipose tissue from 159 obese individuals [46]. Rather, they suggested the possibility that tissue-specific expression of specific isoforms might be important for the functional consequences of TCF7L2-dependent signaling.

In this study, we have provided the evidence for differential expression of long verses medium or short isoforms of TCF7L2 under the nutritional stress in mouse liver. Under insulin resistance, expression levels of the medium and short isoforms of TCF7L2, which reside mostly in the nucleus, are specifically reduced while no such change is observed on that of long isoforms of TCF7L2 in mouse liver. The medium and short isoforms of TCF7L2 lack CtBP binding domain as well as auxiliary DNA binding domain termed C-clamp motif, and have shown to bind to the previously defined TBE sequence [47]. Interestingly, we located putative TBEs at or near the cAMP response element (CRE) or insulin response element (IRE) on the promoters of gluconeogenic genes such as PEPCK and G6Pase (Figure 3A), and found that binding of TCF7L2 inhibited the recruitment of CREB, CRTC2, or FoxO1 on the promoter under feeding conditions in mouse liver or in hepatocytes (Figure 3B and Figure 6D). TCF7L2 *per se* might not directly affect insulin signaling in the liver, since we did not observe any changes in phosphorylation status of key enzymes in hepatic insulin signaling upon knockdown or knockout of TCF7L2, at least under normal chow diet. Rather, we suspected that reduced expression of nuclear TCF7L2 by insulin resistance might be in part responsible for the enhanced hepatic glucose production, providing a potential mechanism for the hyperglycemic phenotype that is induced by DIO or genetic insulin resistance in mammals (Figure 7F). We found that cAMP treatment could reduce expression of TCF7L2 in primary hepatocytes. Interestingly, glucagon/cAMP signaling pathway was known to be induced by insulin resistance in the liver. Further study is necessary to elucidate the potential regulation of TCF7L2 expression or activity by cAMP signaling pathway that is critical in glucose homeostasis *in vivo*.

While we were preparing our manuscript, a new study by Nobrega's group was published suggesting that alterations in TCF7L2 expression would promote changes in glucose metabolism [48]. Surprisingly, they found the seemingly the opposite phenotype on their TCF7L2 null allele compared with our results, in that the TCF7L2 knockout mice displayed hypoglycemia that was associated with reduced plasma insulin levels. As well, systemic overexpression of TCF7L2 rather promoted hyperglycemia in their BAC transgenic models. We suspected the differences between two mouse lines might stem from the fact while we used the C57BL/6 mice for our transient/chronic models, they chose to use CD-1 mice that were rarely utilized for metabolic studies. In addition, while our knockout strategy produced a non-functional protein without the critical DNA binding domain as shown in our study (Figure 3), the null mice designed by Nobrega's group still produced a chimeric protein containing both DNA binding domain and β-catenin binding domain, making it difficult to assess the potential non-specific effect in the cellular signaling pathway driven by the chimeric protein. Furthermore, we employed the hyperinsulinemic-euglycemic clamp techniques to directly measure the endogenous hepatic glucose production as well as whole body glucose metabolism, and directly provided the evidence for the role of TCF7L2 in hepatic glucose production, while they only performed the glucose tolerance test without the further assessment of the role of other tissues that might affect the glucose homeostasis in their mice. Indeed, the role of TCF7L2 in reducing hepatic glucose production in the transformed hepatic cell line was also recently reported [49], supporting our *in vivo* data that alterations in hepatic TCF7L2 expression might be critical in glucose production in the mammalian liver. Given the fact that changes in gluconeogenic gene expression per se might not be enough to invoke changes in hepatic glucose production [50], TCF7L2 might affect yet to be identified pathways to invoke changes in glucose metabolism *in vivo*. Unbiased systemic approaches might be useful to identify potential transcriptional targets of TCF7L2 in this regard.

In summary, we have provided the evidence for the influence of insulin-resistance on the isoform-specific expression of TCF7L2 in the liver, which contributes to the increased glucose production and the resultant hyperglycemia in mammals. A combination of DIO and genetic heterozygous mutations is considered a critical risk factor for the development of type 2 diabetes. DIO-mediated or genetic haploinsufficiency of TCF7L2 promotes hyperglycemia and insulin resistance in mouse models, suggesting that dysregulation of TCF7L2 expression in the liver might be a critical contributor for the insulin resistance and hyperglycemia in humans. Further study is necessary to provide the link between the differential expression patterns for TCF7L2 in the liver and the progression of diabetes in the affected patients.

## Materials and Methods

### Plasmids

Full-length sequence of TCF7L2 was PCR-amplified from pYX-mouse TCF7L2 (Invitrogen), and was subcloned into pcDNA3-FLAG. TCF7L2 isoforms (TCF7L2 M, S, and E), TCF7L2 M mutants (Δβ-catenin and ΔHMG), and β-catenin were generated using site-directed mutagenesis. To generate pU6-TCF7L2 RNAi, palindromic sequences corresponding to nucleotides 773–798 from mouse TCF7L2 coding sequence (5′-CCA CAG CGC TGA CAG TCA ACG CAT CT-3′) were linked to human U6 promoter in the pBluescript KS vector (Stratagene). hG6Pase (−1227/+57) Luc and PEPCK Luc were generated based on the previous report [51].

### Recombinant adenoviruses

Adenoviruses expressing GFP only, nonspecific RNAi control (US), and CRTC2 were described previously [12]. Adenovirus expressing TCF7L2 isoforms, TCF7L2 mutants, TCF7L2 RNAi, FoxO1, FoxO1 RNAi, β-catenin, or β-catenin RNAi were generated by homologous recombination between adenovirus backbone vector pAD-Easy and linearized transfer vector pAD-Track as described previously [52]. For animal experiments, viruses were purified on a CsCl gradient, dialyzed against PBS buffer containing 10% glycerol, and stored at −80°C.

### Animal experiments

Male 4 or 7-week-old C57BL/6 mice were purchased form ORIENT BIO. TCF7L2 heterozygous null mice (*TCF7L2*+/−) were obtained from EUCOMM consortium and were backcrossed with C57BL/6 for 5 times before being used for the experiment. Mice were housed in a specific pathogen-free animal facility at the Sungkyunkwan University School of Medicine (12∶12 h light-dark cycle). To induce obesity and insulin resistance, male 4-week-old mice were fed a high-fat diet (60 kcal % fat diet: D12492 of Research Diets) for 8–10 weeks. For animal experiments involving adenoviruses, mice were tail vein-injected with recombinant adenovirus (0.1–0.5×10^9^ pfu per mice). Adenovirus-mediated expression was exclusively detected in the liver tissues, but not in other insulin sensitive tissues (data not shown). In addition, plasma ALT and AST levels were not significantly different between mice among the same experimental groups that were injected with various adenoviruses (data not shown). To measure fasting blood glucose level, animals were fasted for 16 h or 6 h with free access to water. For glucose tolerance test (GTT) and pyruvate tolerance test (PTT), 16 h-fasted mice were injected intraperitoneally with glucose (2 g/kg of body weight for chow diet and 1.5 g/kg of body weight for high-fat diet). For insulin tolerance test (ITT), 6 h-fasted mice were injected intraperitoneally with 1 unit/kg (chow diet) or 1.5 unit/kg (high-fat diet) body weight of insulin. Blood glucose levels were measured from tail vein blood collected at the designated times. All procedures were approved by the Sungkyunkwan University School of Medicine Institutional Animal Care and Use Committee (IACUC).

### Culture of primary hepatocytes and measurement of glucose production

Primary hepatocytes were isolated from 200 g of Sprague Dawley rats or 8-week-old male C57BL/6 mice by collagenase perfusion method [12]. Briefly, 1×10^6^ cells were plated in 6-well plates with medium 199 (Sigma) supplemented by 10% FBS, 10 units/ml penicillin, 10 µg/ml streptomycin, and 10 nM dexamethasone for 6 h. After attachment, cells were infected with adenovirus for 24 h (for adenovirus expressing GFP, TCF7L2 M, TCF7L2 S, TCF7L2 E, CRTC2, or FoxO1) or 48 h (for adenovirus expressing US, TCF7L2 RNAi, β-catenin RNAi, CRTC2 RNAi, or FoxO1 RNAi). Subsequently, cells were maintained in medium 199 without 10% FBS for 18 h, and were treated with 10 µM forskolin for 2 h or 100 nM insulin for 24 h (for RNA) and 15 min (for protein). To measure glucose production, cells were incubated in serum-free media for 16 h, and then were stimulated with 10 µM forskolin and 1 nM dexamethasone and/or 100 nM insulin in Krebs-ringer buffer containing gluconeogenic substrates (20 mM lactate and 2 mM pyruvate) for 8 h. Glucose concentrations were measured using a Glucose Assay Kit (Cayman Chemical).

### Quantitative PCR

Total RNA from either primary hepatocytes or liver tissue was extracted using Easy-spin total RNA extract kit (iNtRON biotechnology, Inc.). 1 µg of total RNA was used for generating cDNA with amfiRivert reverse transcriptase (GenDEPOT), and was analyzed by quantitative PCR using SYBR green PCR kit and TP800 Thermal Cycler Dice Real Time System (TAKARA). PCR array for glucose metabolism was purchased from Qiagen, and was used according to the manufacturer's instructions. All data were normalized to expression of ribosomal *L32* in the corresponding sample.

### Transfection assays

Human hepatoma HepG2 cells were maintained with Ham's F12 medium supplemented with 10% FBS, 10 units/ml penicillin, and 10 µg/ml streptomycin. For transfection, TrnasIT-LT1 Reagent (Mirus Bio Corporation) was used according to the manufacturer's instructions. Each transfection was performed with 200 ng of luciferase construct, 50 ng of β-galactosidase plasmid, and 2.5–10 ng of expression vector for TCF7L2 M, TCF7L2 S, TCF7L2 E, CRTC2, or FOXO1. After 24 h, cells were serum starved for 18 h, and then were stimulated with either 10 µM forskolin or DMSO vehicle for 4 h.

### Western blot analyses

Western blot analyses of whole-cell extracts were performed as described [53]. The specific primary antisera for TCF7L2 M, S, and E were produced from GenScript. Antibodies for TCF7L2, AKT, phosphor-AKT, phosphor-GSK3β, FOXO1, and phosphor-FOXO1 were from Cell Signaling Technology. Antibodies for HSP90, insulin receptor, and GSK3β were obtained from Santa Cruz, antibodies for α-tubulin, β-actin, and flag-M2 were provided from Sigma-Aldrich, antibody for CRTC2 was from Calbiochem, and antibody for phospho-insulin receptor (Tyr1162/1163) was from Millipore. The specific signals were amplified by addition of horseradish peroxidase-conjugated secondary antibodies (Abcam), and were visualized by using an enhanced chemiluminescence system (Abfrontier).

### Chromatin immunoprecipitation

Nuclear isolation, cross-linking, and chromatin immunoprecipitation assays on mouse primary hepatocyte samples were performed as described previously (Jaeschke and Davis, 2007). Precipitated DNA fragments were analyzed by PCR using primers against relevant mouse promoters.

### Measurement of metabolites

Blood glucose levels were determined from tail vein blood using an automatic glucose monitor (One Touch; LifeScan, Inc.). Plasma TG and NEFA were measured by colorimetric assay kits (Wako). Plasma insulin was measured by Mouse Insulin ELISA Kit (U-Type; Shibayagi Corp.). Plasma IGFBP1 was measured by Mouse IGFBP-1 ELISA Kit (Immuno-biological Laboratories, Inc.). Hepatic glycogen level was measured by EnzyChrom Glycogen Assay Kit (BioAssay Systems). Total liver lipids were extracted with chloroform-methanol (2∶1, v/v) mixture as described previously [54].

### Hyperinsulinemic-euglycemic clamp study

Seven days prior to the hyperinsulinemic-euglycemic clamp studies, indwelling catheters were placed into the right internal jugular vein extending to the right atrium. After an overnight fast, [3-^3^H]glucose (HPLC purified; PerkinElmer) was infused at a rate of 0.05 mCi/min for 2 h to assess the basal glucose turnover. Following the basal period, hyperinsulinemic-euglycemic clamp was conducted for 120 min with a primed/continuous infusion of human insulin (84 pmol/kg prime, and 12 pmol/kg/min infusion) (Eli Lilly). Blood samples (10 ml) were collected at 10–20 min intervals, plasma glucose was immediately analyzed during the clamps by a glucose oxidase method (GM9 Analyzer; Analox Instruments), and 20% dextrose was infused at variable rates to maintain plasma glucose at basal concentrations (6.7 mM). To estimate insulin-stimulated whole-body glucose fluxes, [3-^3^H]glucose was infused at a rate of 0.1 mCi/min throughout the clamps as previously described [55], [56]. Blood samples (10 ml) for the measurement of plasma ^3^H activity were taken at the end of the basal period and during the last 45 min of the clamp. Glucose flux was calculated as described previously [55], [56].

### Statistical analysis

Results of Q-PCR and promoter assay were shown as mean ± SD. Values of metabolites were shown as mean ± SEM. The comparison of different groups was performed using two-tailed unpaired Student's t test. In all statistical comparisons, *p* value<0.05 were considered statistically significant and reported as in legends.

## Supporting Information

Figure S1 Expression and cellular distribution of hepatic TCF7L2. A) Western blot analysis showing protein expression levels of TCF7L2 M, TCF7L2 S, and TCF7L2 E in livers of fasted or fed mice. B) Western blot analysis showing endogenous localization of TCF7L2 variants in mouse primary hepatocytes (W; whole cell lysates, N; nuclear fraction, C; cytoplasmic fraction). Representative data from at least three independent experiments are shown. C–D) Western blot analysis and Q-PCR analysis showing protein and mRNA expression levels of TCF7L2 by treatment of Insulin (C) or forskolin (D). Representative data from at least three independent experiments are shown. Data in C) and D) represent mean ± SD (*;*P*<0.05, **;*P*<0.005, ***;*P*<0.0005, t-test).
(TIF)

Figure S2 Effect of TCF7L2 knockdown on glucose metabolism in the liver. A) Immunohistochemistry data showing the effect of knockdown by Ad-shTCF7L2 in mouse liver. Representative data are shown (*n* = 7–8 each). B) and C) Body weight changes and plasma insulin level (B), Liver TG, plasma TG, and plasma NEFA levels (C) from 8-week-old C57BL/6 male mice that were infected with Ad-US (*n* = 7) or Ad-shTCF7L2 (*n* = 6). D) Body weight changes, serum IGFBP1, and serum insulin levels from 8-week-old C57BL/6 male mice that were infected with Ad-US (*n* = 5) or Ad-shTCF7L2 (*n* = 5) under feeding conditions. E) Glucose tolerance test showing effects of Ad-shTCF7L2 from 8-week-old C57BL/6 male mice that were infected with Ad-US (*n* = 7) or Ad-shTCF7L2 (*n* = 6). F) Insulin tolerance test showing effects of Ad-shTCF7L2 on insulin signaling pathway in mice (*n* = 7 for Ad-US, and *n* = 6 for Ad-shTCF7L2). Data in B–F) represent mean ± SEM (*;*P*<0.05, ***;*P*<0.0005, t-test).
(TIF)

Figure S3 Effects of TCF7L2 depletion on hepatic gluconeogenic program. A) Western blot analysis showing effects of Ad-shTCF7L2 on insulin signaling in mouse liver under feeding conditions. B) Q-PCR analysis showing effects of Ad-shTCF7L2 on gluconeogenic gene expression in mouse primary hepatocytes. Representative data from at least three independent experiments are shown. C) Western blot analysis showing effects of TCF7L2 expression on insulin signaling in mouse primary hepatocytes. Cells were treated with 100 nM insulin for 15 min before being harvested. Representative data from at least three independent experiments are shown. D) Western blot showing change in protein expression level of TCF7L2 by treatment of Insulin (12 h) or forskolin (2 h). Representative data from at least three independent experiments are shown. E) Q-PCR analysis showing effect of forskolin (2 h) or insulin (12 h) on G6Pase mRNA level in mouse primary hepatocytes infected with Ad-shTCF7L2. Representative data from at least three independent experiments are shown. Data in B) and E) represent mean ± SD (*;*P*<0.05, **;*P*<0.005, ***;*P*<0.0005, t-test).
(TIF)

Figure S4 Effects of TCF7L2 expression on hepatic gluconeogenic program. A) Western blot analysis (left) and Q-PCR analysis (right) showing effects of splicing variants of TCF7L2 on expression levels of gluconeogenic genes in mouse primary hepatocytes (*n* = 3 for each group). Representative data from at least three independent experiments are shown. B) Body weight changes from high-fat diet-fed 14-week-old C57BL/6 male mice that were infected with Ad-GFP (*n* = 10) or Ad-TCF7L2 M adenovirus (*n* = 7). C) 6 h fasting plasma insulin levels from high-fat diet-fed 14-week-old C57BL/6 male mice that were infected with Ad-GFP (*n* = 10) or Ad-TCF7L2 M adenovirus (*n* = 7). D) Insulin tolerance test showing effects of TCF7L2 expression on glucose homeostasis (*n* = 8 for Ad-GFP, and *n* = 7 for Ad-TCF7L2 M). E) Glucose output assay showing effects of TCF7L2 expression on glucose production in primary hepatocytes (*n* = 3 for each group). Representative data from at least three independent experiments are shown. F) Western blot analysis showing effects of TCF7L2 expression on insulin signaling in primary hepatocytes. Cells were treated with 100 nM insulin for 15 min before being harvested. Representative data from at least three independent experiments are shown. G) Transfection analysis was performed to determine the effects of TCF7L2 isoforms on CRTC2- or FOXO1a-dependent activation of G6Pase and PEPCK promoter activities in HepG2 cells (*n* = 3 for each group). Representative data from at least three independent experiments are shown. Data in A), and G) represent mean ± SD, and data in B–E) represent mean ± SEM (*;*P*<0.05, **;*P*<0.005, ***;*P*<0.0005, t-test).
(TIF)

Figure S5 The role of TCF7L2 expression on gluconeogenic promoter occupancy. A) Transfection analysis showing effects of TCF7L2 expression on promoter activities of wild type or TBE mutants of G6Pase and PEPCK in HepG2 cells (*n* = 3 for each group). Representative data from at least three independent experiments are shown. B) A schematic diagram of a pair of TCF7L2 mutants that is either defective in interacting with β-catenin (Δβ-catenin) or defective in DNA-binding (ΔHMG). C) Co-immunoprecipitation assay showing the physical interaction between TCF7L2 (WT and mutants) and β-catenin. Representative data from at least three independent experiments are shown. D) Chromatin immunoprecipitation experiments showing effects of CRTC2 or FoxO1 on occupancies of TCF7L2 (wild type and mutants) over G6Pase and PEPCK promoters in mouse primary hepatocytes. Representative data from at least three independent experiments are shown. Data in A) and D) represent mean ± SD (**;*P*<0.005, ***;*P*<0.0005, t-test).
(TIF)

Figure S6 Impacts of chronic depletion of TCF7L2 on hepatic glucose production. A) A targeting strategy for critical exons of TCF7L2 was shown . B) 16 h fasting insulin levels from 8-week-old *TCF7L2 +/+* (*n* = 7) and *TCF7L2 +/−* (*n* = 6) male mice under the normal chow diet were shown. C) Western blot analysis showing relative expression of TCF7L2 isoforms in livers of *TCF7L2 +/+* mice and *TCF7L2 +/−* mice. D) Glucose tolerance test (upper) and insulin secretion at 15 min post-glucose injection (bottom) showing effects of chronic depletion of TCF7L2 on glucose homeostasis (*n* = 5 for each group). E) Liver glycogen level from 8-week-old *TCF7L2 +/+* (*n* = 7) and *TCF7L2 +/−* (*n* = 7) male mice under the feeding condition. F) Peripheral and hepatic glucose metabolism was assessed by means of hyperinsulinemic-euglycemic clamps (*n* = 7 for *TCF7L2* +/+ mice, and *n* = 5 for *TCF7L2* +/− mice). From left to right, basal and clamp hepatic glucose production, rates of glucose turnover, rates of whole body glycolysis, and rates of whole body glycogen synthesis are shown. G) Effects of haploinsufficiency of TCF7L2 on body weight, fat mass, and lean mass during the hyperinsulinemic-euglycemic clamp study (*n* = 10 for *TCF7L2 +/+* mice, and *n* = 7 for *TCF7L2 +/−* mice). Data in B) and D–G) represent mean ± SEM (*;*P*<0.05, **;*P*<0.005, ***;*P*<0.0005, t-test).
(TIF)

Figure S7 Effects of chronic depletion of TCF7L2 on insulin signaling pathway in mice. A–D) Western blot analysis showing insulin signaling in the liver (A), pancreas (B), adipose tissues (C), or skeletal muscle (D) of *TCF7L2 +/+* and *TCF7L2 +/−* mice following an acute injection of a bolus of insulin (10 min).
(TIF)

Figure S8 Effects of CRTC2 and/or FoxO1 knockdown with chronic depletion of TCF7L2 in primary hepatocytes. A) Western blot analysis showing depletion of CRTC2 and FoxO1 in primary hepatocytes from *TCF7L2 +/+* and *TCF7L2 +/−* mice. Representative data from at least three independent experiments are shown. B) Q-PCR analysis showing effects of Ad-shCRTC2 and Ad-shFoxO1 on gluconeogenic gene expression in primary hepatocytes from *TCF7L2 +/+* and *TCF7L2 +/−* mice. Representative data from at least three independent experiments are shown. Data in B) represent mean ± SD (**;*P*<0.005, ***;*P*<0.0005, t-test).
(TIF)
