# Supplementary figures and images for: TCF7L2 Modulates Glucose Homeostasis by Regulating CREB- and FoxO1-Dependent Transcriptional Pathway in the Liver
Source: PLoS Genet. 2012 Sep 27;8(9):e1002986. doi: 10.1371/journal.pgen.1002986 (PMC3459990; doi:10.1371/journal.pgen.1002986)

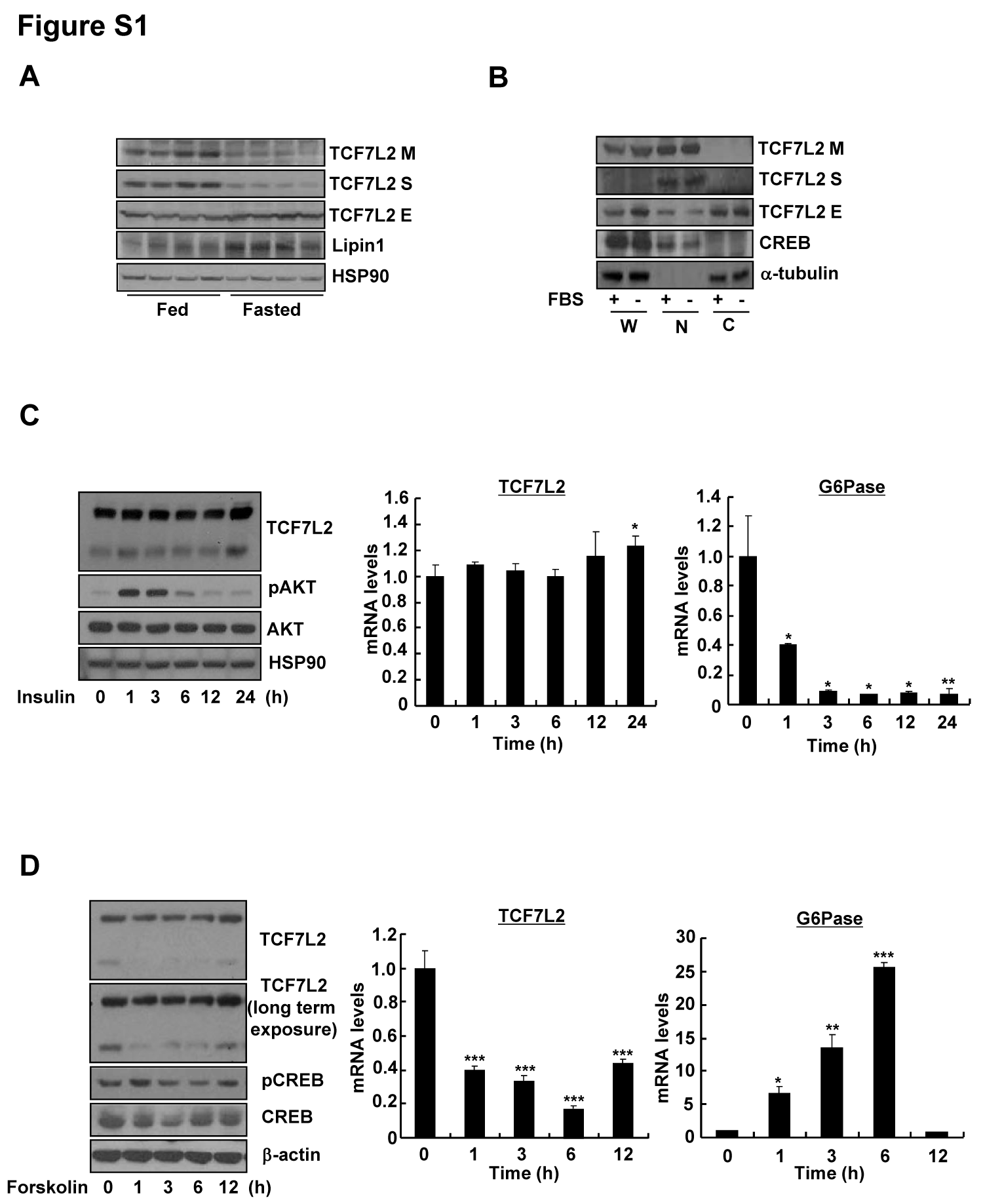

Supplement: Figure S1 — Expression and cellular distribution of hepatic TCF7L2. A) Western blot analysis showing protein expression levels of TCF7L2 M, TCF7L2 S, and TCF7L2 E in livers of fasted or fed mice. B) Western blot analysis showing endogenous localization of TCF7L2 variants in mouse primary hepatocytes (W; whole cell lysates, N; nuclear fraction, C; cytoplasmic fraction). Representative data from at least three independent experiments are shown. C–D) Western blot analysis and Q-PCR analysis showing protein and mRNA expression levels of TCF7L2 by treatment of Insulin (C) or forskolin (D). Representative data from at least three independent experiments are shown. Data in C) and D) represent mean ± SD (*;P<0.05, **;P<0.005, ***;P<0.0005, t-test). (TIF) [file pgen.1002986.s001.tif]

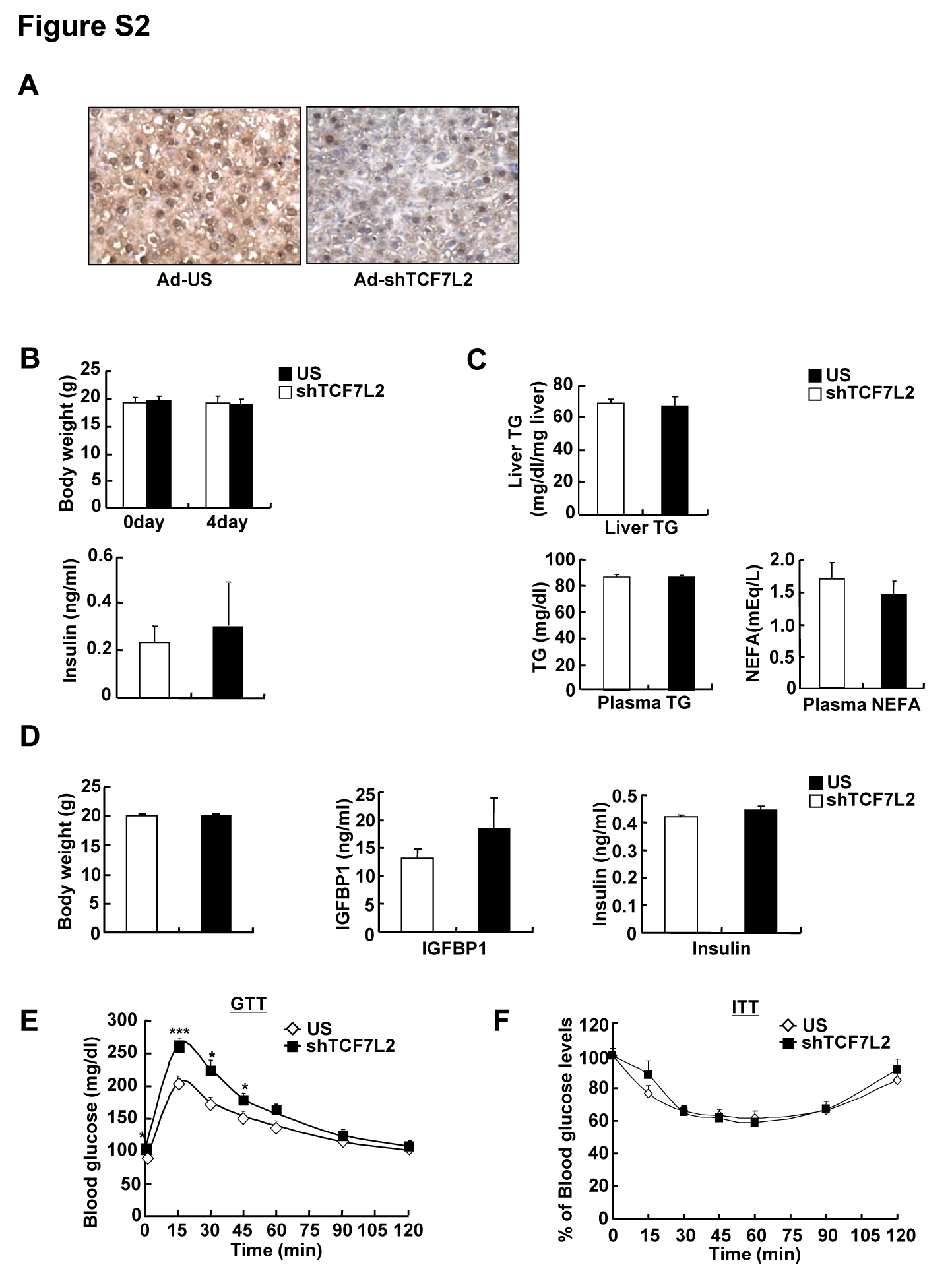

Supplement: Figure S2 — Effect of TCF7L2 knockdown on glucose metabolism in the liver. A) Immunohistochemistry data showing the effect of knockdown by Ad-shTCF7L2 in mouse liver. Representative data are shown (n = 7–8 each). B) and C) Body weight changes and plasma insulin level (B), Liver TG, plasma TG, and plasma NEFA levels (C) from 8-week-old C57BL/6 male mice that were infected with Ad-US (n = 7) or Ad-shTCF7L2 (n = 6). D) Body weight changes, serum IGFBP1, and serum insulin levels from 8-week-old C57BL/6 male mice that were infected with Ad-US (n = 5) or Ad-shTCF7L2 (n = 5) under feeding conditions. E) Glucose tolerance test showing effects of Ad-shTCF7L2 from 8-week-old C57BL/6 male mice that were infected with Ad-US (n = 7) or Ad-shTCF7L2 (n = 6). F) Insulin tolerance test showing effects of Ad-shTCF7L2 on insulin signaling pathway in mice (n = 7 for Ad-US, and n = 6 for Ad-shTCF7L2). Data in B–F) represent mean ± SEM (*;P<0.05, ***;P<0.0005, t-test). (TIF) [file pgen.1002986.s002.tif]

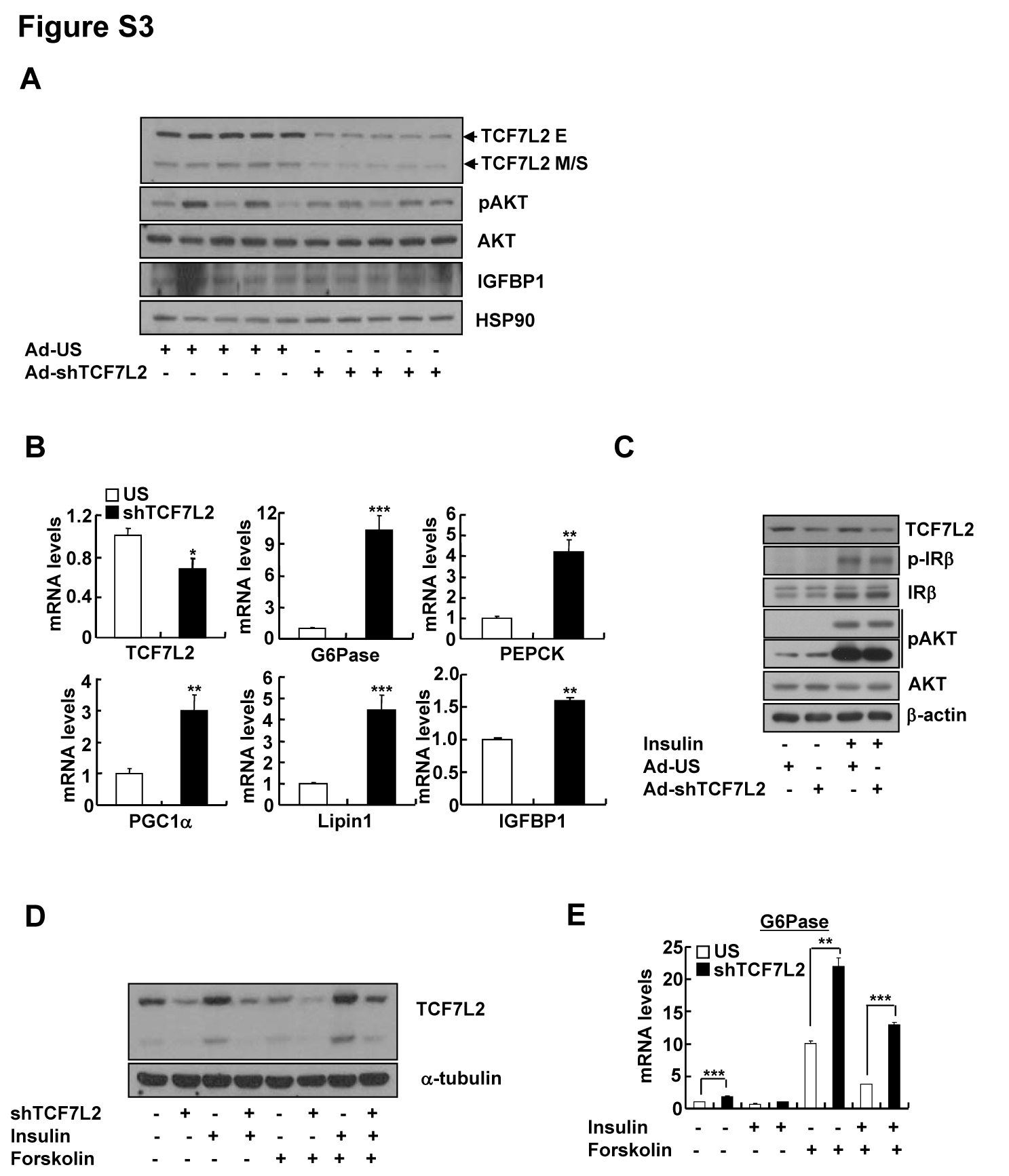

Supplement: Figure S3 — Effects of TCF7L2 depletion on hepatic gluconeogenic program. A) Western blot analysis showing effects of Ad-shTCF7L2 on insulin signaling in mouse liver under feeding conditions. B) Q-PCR analysis showing effects of Ad-shTCF7L2 on gluconeogenic gene expression in mouse primary hepatocytes. Representative data from at least three independent experiments are shown. C) Western blot analysis showing effects of TCF7L2 expression on insulin signaling in mouse primary hepatocytes. Cells were treated with 100 nM insulin for 15 min before being harvested. Representative data from at least three independent experiments are shown. D) Western blot showing change in protein expression level of TCF7L2 by treatment of Insulin (12 h) or forskolin (2 h). Representative data from at least three independent experiments are shown. E) Q-PCR analysis showing effect of forskolin (2 h) or insulin (12 h) on G6Pase mRNA level in mouse primary hepatocytes infected with Ad-shTCF7L2. Representative data from at least three independent experiments are shown. Data in B) and E) represent mean ± SD (*;P<0.05, **;P<0.005, ***;P<0.0005, t-test). (TIF) [file pgen.1002986.s003.tif]

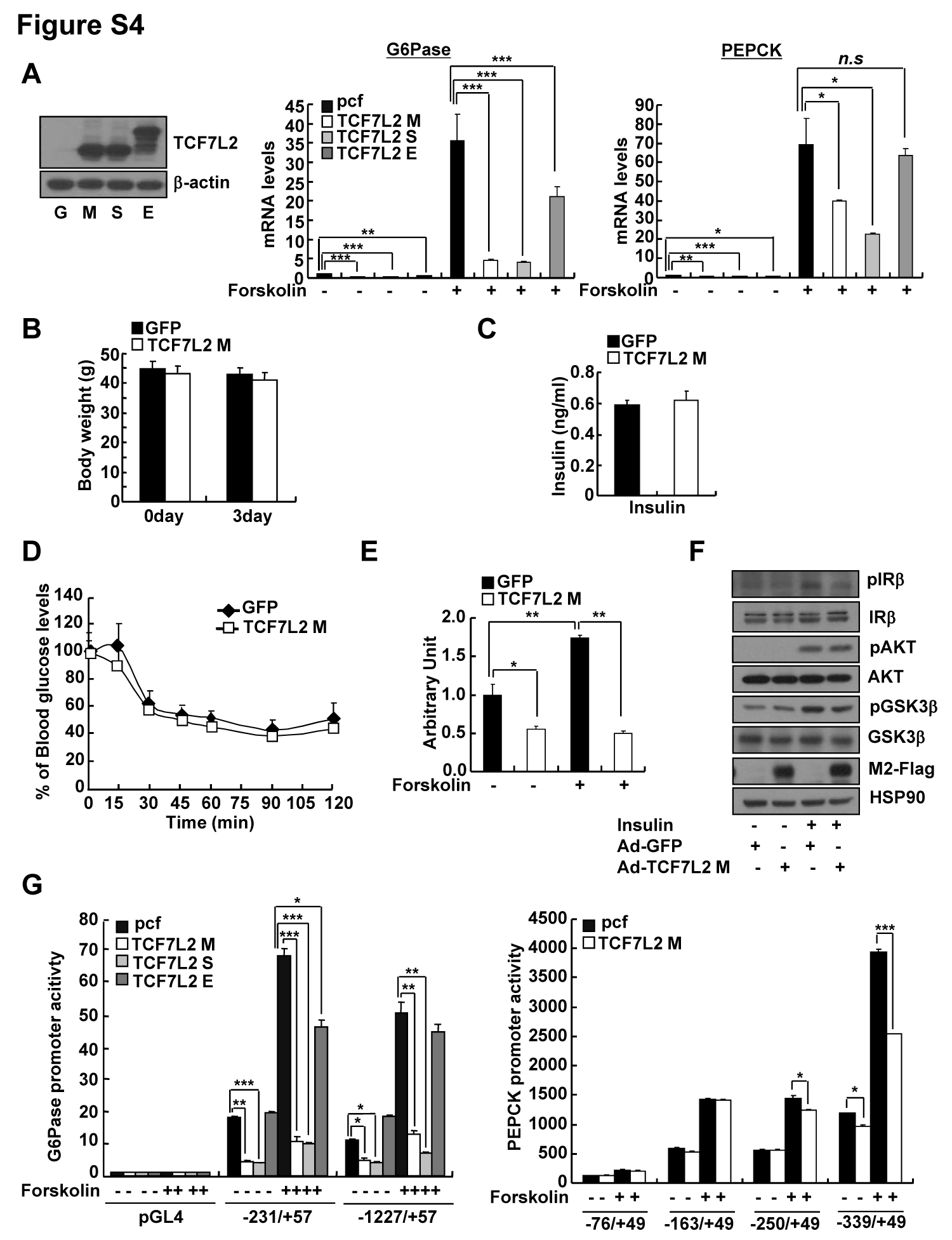

Supplement: Figure S4 — Effects of TCF7L2 expression on hepatic gluconeogenic program. A) Western blot analysis (left) and Q-PCR analysis (right) showing effects of splicing variants of TCF7L2 on expression levels of gluconeogenic genes in mouse primary hepatocytes (n = 3 for each group). Representative data from at least three independent experiments are shown. B) Body weight changes from high-fat diet-fed 14-week-old C57BL/6 male mice that were infected with Ad-GFP (n = 10) or Ad-TCF7L2 M adenovirus (n = 7). C) 6 h fasting plasma insulin levels from high-fat diet-fed 14-week-old C57BL/6 male mice that were infected with Ad-GFP (n = 10) or Ad-TCF7L2 M adenovirus (n = 7). D) Insulin tolerance test showing effects of TCF7L2 expression on glucose homeostasis (n = 8 for Ad-GFP, and n = 7 for Ad-TCF7L2 M). E) Glucose output assay showing effects of TCF7L2 expression on glucose production in primary hepatocytes (n = 3 for each group). Representative data from at least three independent experiments are shown. F) Western blot analysis showing effects of TCF7L2 expression on insulin signaling in primary hepatocytes. Cells were treated with 100 nM insulin for 15 min before being harvested. Representative data from at least three independent experiments are shown. G) Transfection analysis was performed to determine the effects of TCF7L2 isoforms on CRTC2- or FOXO1a-dependent activation of G6Pase and PEPCK promoter activities in HepG2 cells (n = 3 for each group). Representative data from at least three independent experiments are shown. Data in A), and G) represent mean ± SD, and data in B–E) represent mean ± SEM (*;P<0.05, **;P<0.005, ***;P<0.0005, t-test). (TIF) [file pgen.1002986.s004.tif]

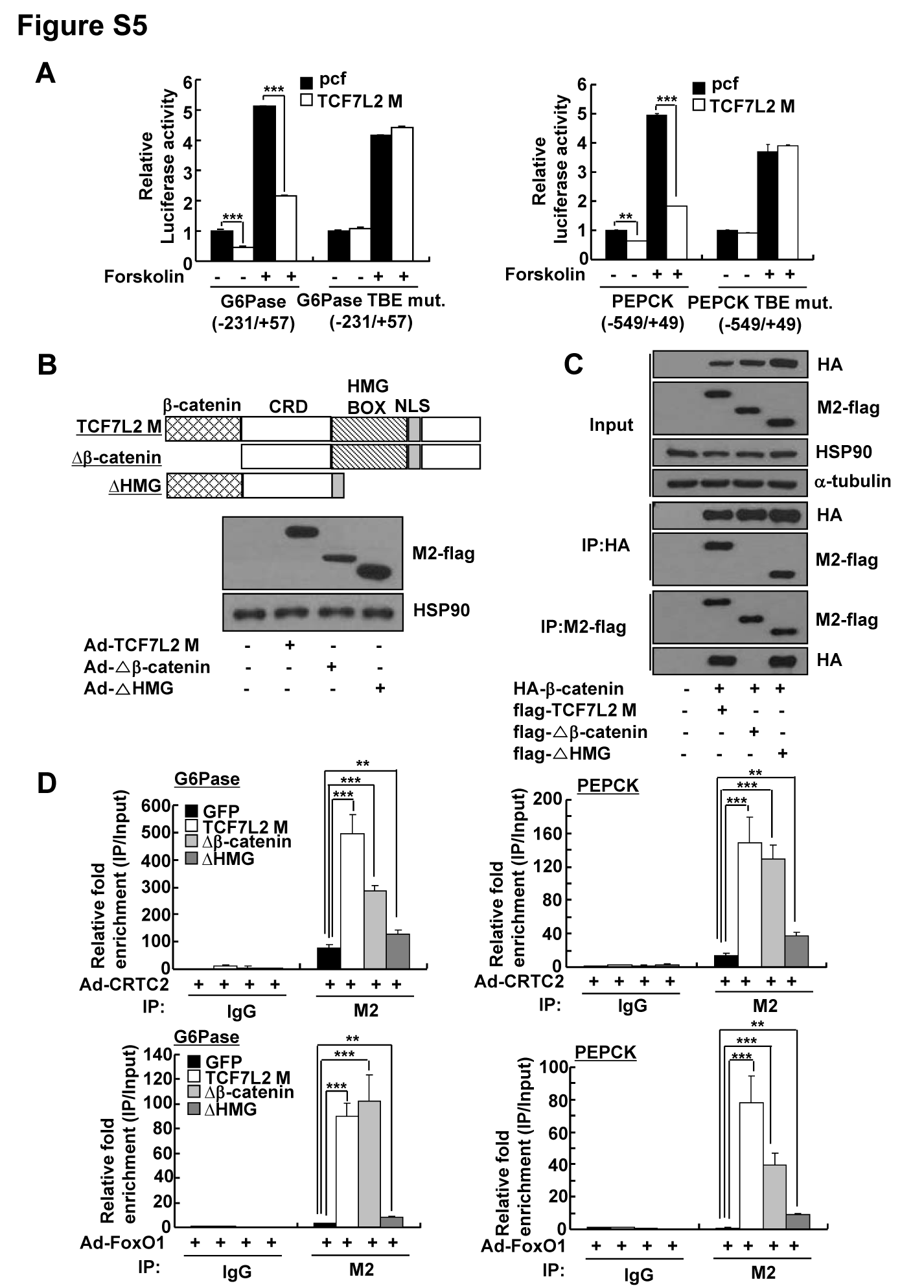

Supplement: Figure S5 — The role of TCF7L2 expression on gluconeogenic promoter occupancy. A) Transfection analysis showing effects of TCF7L2 expression on promoter activities of wild type or TBE mutants of G6Pase and PEPCK in HepG2 cells (n = 3 for each group). Representative data from at least three independent experiments are shown. B) A schematic diagram of a pair of TCF7L2 mutants that is either defective in interacting with β-catenin (Δβ-catenin) or defective in DNA-binding (ΔHMG). C) Co-immunoprecipitation assay showing the physical interaction between TCF7L2 (WT and mutants) and β-catenin. Representative data from at least three independent experiments are shown. D) Chromatin immunoprecipitation experiments showing effects of CRTC2 or FoxO1 on occupancies of TCF7L2 (wild type and mutants) over G6Pase and PEPCK promoters in mouse primary hepatocytes. Representative data from at least three independent experiments are shown. Data in A) and D) represent mean ± SD (**;P<0.005, ***;P<0.0005, t-test). (TIF) [file pgen.1002986.s005.tif]

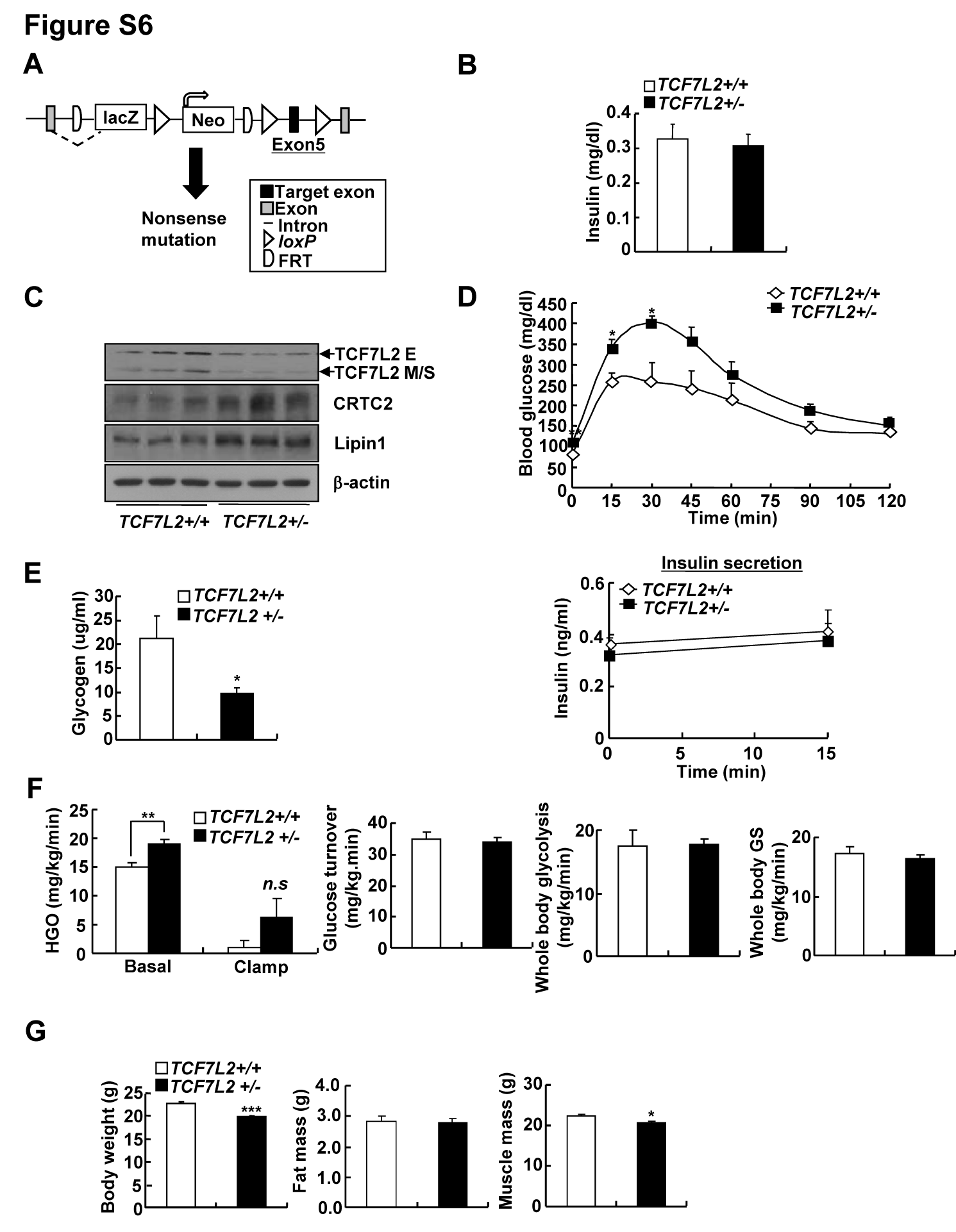

Supplement: Figure S6 — Impacts of chronic depletion of TCF7L2 on hepatic glucose production. A) A targeting strategy for critical exons of TCF7L2 was shown . B) 16 h fasting insulin levels from 8-week-old TCF7L2 +/+ (n = 7) and TCF7L2 +/− (n = 6) male mice under the normal chow diet were shown. C) Western blot analysis showing relative expression of TCF7L2 isoforms in livers of TCF7L2 +/+ mice and TCF7L2 +/− mice. D) Glucose tolerance test (upper) and insulin secretion at 15 min post-glucose injection (bottom) showing effects of chronic depletion of TCF7L2 on glucose homeostasis (n = 5 for each group). E) Liver glycogen level from 8-week-old TCF7L2 +/+ (n = 7) and TCF7L2 +/− (n = 7) male mice under the feeding condition. F) Peripheral and hepatic glucose metabolism was assessed by means of hyperinsulinemic-euglycemic clamps (n = 7 for TCF7L2 +/+ mice, and n = 5 for TCF7L2 +/− mice). From left to right, basal and clamp hepatic glucose production, rates of glucose turnover, rates of whole body glycolysis, and rates of whole body glycogen synthesis are shown. G) Effects of haploinsufficiency of TCF7L2 on body weight, fat mass, and lean mass during the hyperinsulinemic-euglycemic clamp study (n = 10 for TCF7L2 +/+ mice, and n = 7 for TCF7L2 +/− mice). Data in B) and D–G) represent mean ± SEM (*;P<0.05, **;P<0.005, ***;P<0.0005, t-test). (TIF) [file pgen.1002986.s006.tif]

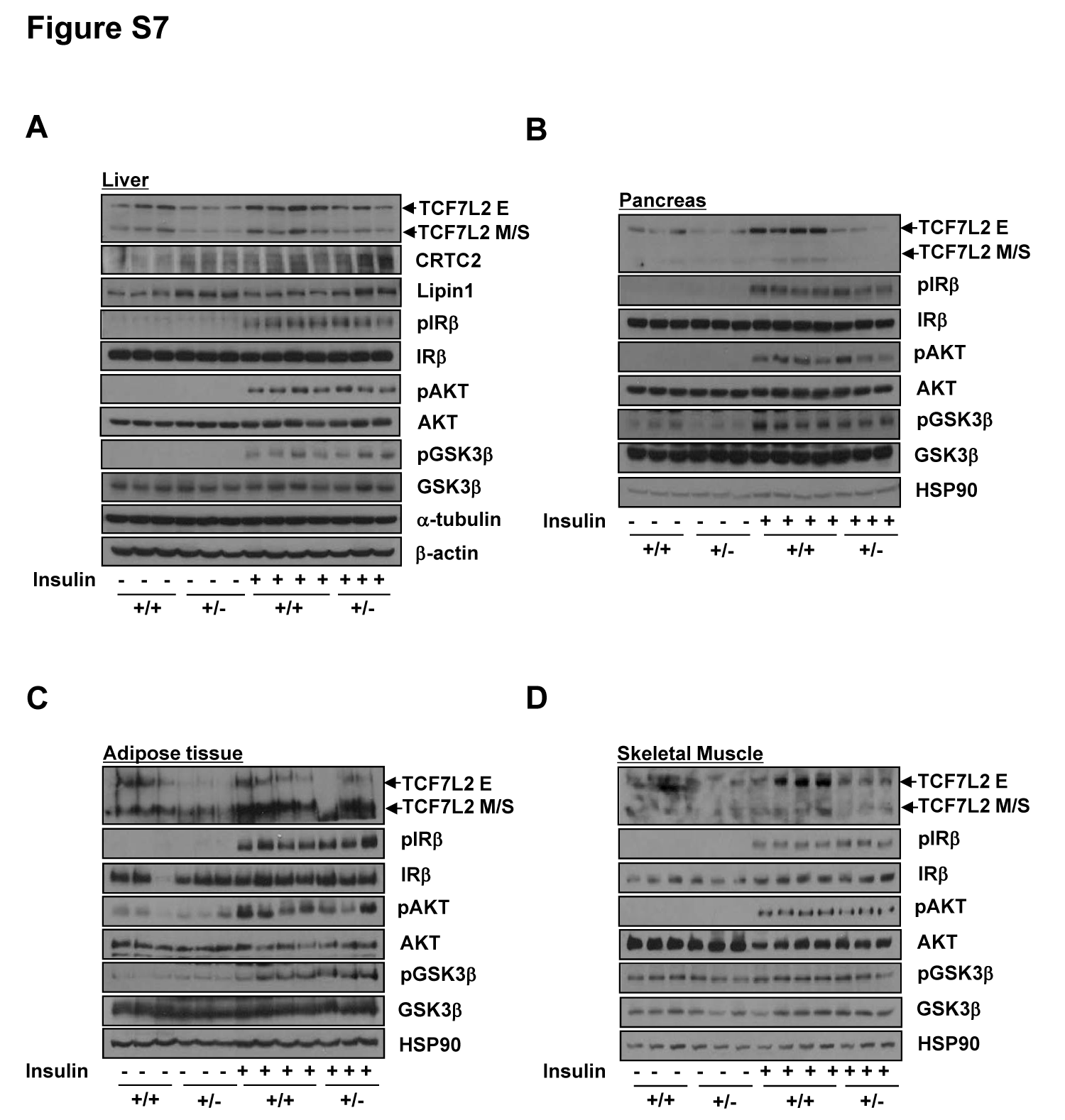

Supplement: Figure S7 — Effects of chronic depletion of TCF7L2 on insulin signaling pathway in mice. A–D) Western blot analysis showing insulin signaling in the liver (A), pancreas (B), adipose tissues (C), or skeletal muscle (D) of TCF7L2 +/+ and TCF7L2 +/− mice following an acute injection of a bolus of insulin (10 min). (TIF) [file pgen.1002986.s007.tif]

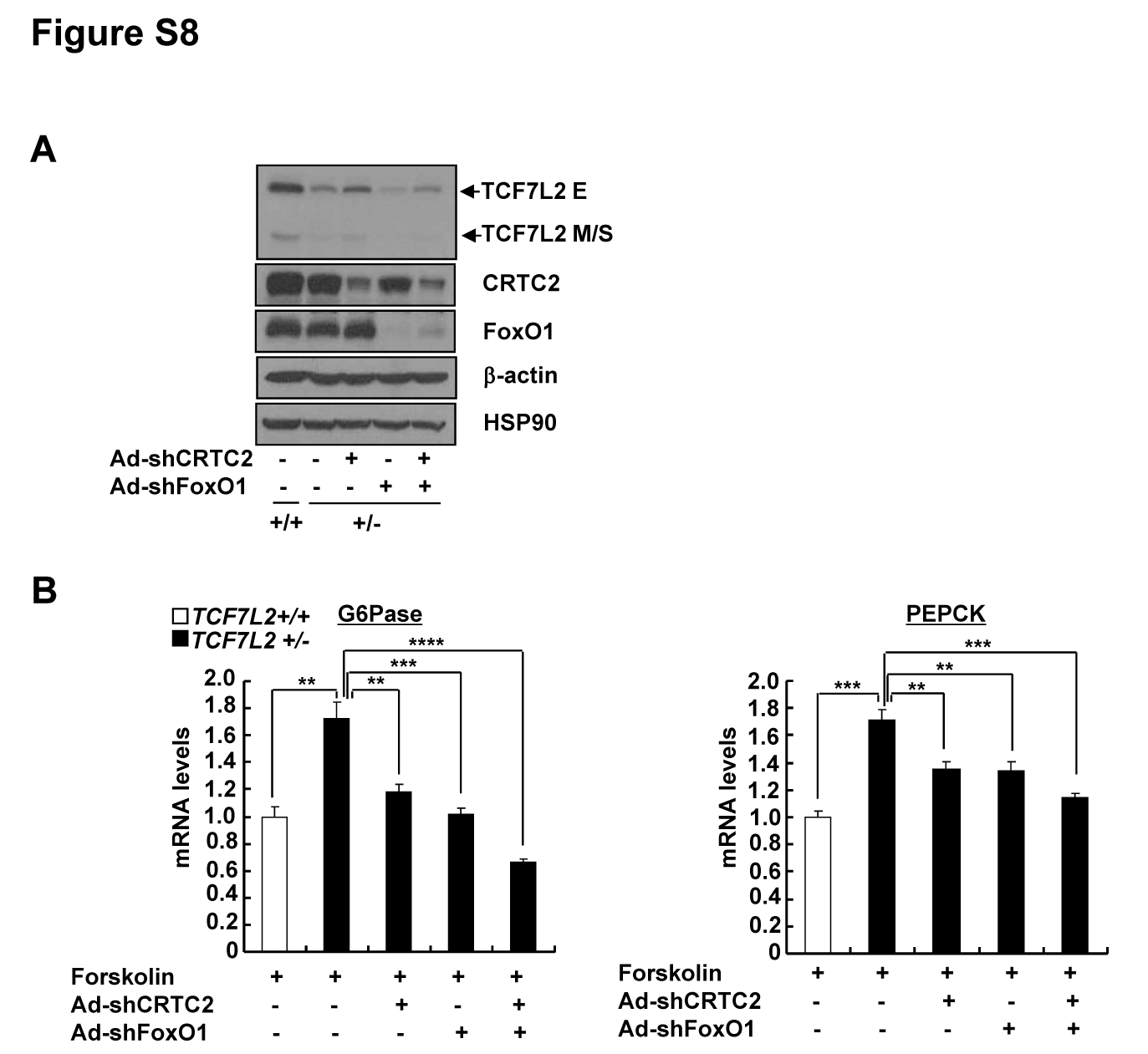

Supplement: Figure S8 — Effects of CRTC2 and/or FoxO1 knockdown with chronic depletion of TCF7L2 in primary hepatocytes. A) Western blot analysis showing depletion of CRTC2 and FoxO1 in primary hepatocytes from TCF7L2 +/+ and TCF7L2 +/− mice. Representative data from at least three independent experiments are shown. B) Q-PCR analysis showing effects of Ad-shCRTC2 and Ad-shFoxO1 on gluconeogenic gene expression in primary hepatocytes from TCF7L2 +/+ and TCF7L2 +/− mice. Representative data from at least three independent experiments are shown. Data in B) represent mean ± SD (**;P<0.005, ***;P<0.0005, t-test). (TIF) [file pgen.1002986.s008.tif]
